# Mortality, Health Care Burden, and Treatment of CKD: A Multinational, Observational Study (OPTIMISE-CKD)

**DOI:** 10.34067/KID.0000000000000374

**Published:** 2024-02-01

**Authors:** Navdeep Tangri, Maria K. Svensson, Johan Bodegård, Samuel Adamsson Eryd, Marcus Thuresson, Stefan Gustafsson, Tadashi Sofue

**Affiliations:** 1 University of Manitoba Max Rady College of Medicine, Winnipeg, Manitoba, Canada; 2 Department of Medical Sciences, Renal Medicine, Uppsala University, Uppsala, Sweden; 3 Uppsala Clinical Research Centre, Uppsala, Sweden; 4 Cardiovascular, Renal and Metabolism Evidence, BioPharmaceuticals Medical, AstraZeneca, Gothenburg, Sweden; 5 Statisticon AB, Uppsala, Sweden; 6 Sence Research AB, Uppsala, Sweden; 7 Department of Cardiorenal and Cerebrovascular Medicine, Kagawa University, Kagawa, Japan

**Keywords:** cardiovascular disease, CKD, diabetes mellitus, economic impact, heart failure, hospitalization, mortality risk

## Abstract

**Key Points:**

- Newly detected, moderately progressed CKD is associated with high clinical risks and health care costs.
- Most patients with moderately progressed CKD do not have diabetes and are at the same clinical risk as those with diabetes.
- Substantial inertia with kidney-protective treatment is observed when moderately progressed CKD is detected.

**Background:**

Kidney-protective treatments (renin–angiotensin system inhibitors and sodium–glucose cotransporter-2 inhibitors [SGLT-2is]) can delay CKD progression, cardiovascular events, and death.

**Methods:**

This observational cohort study used electronic health records and claims data from Japan, Sweden, and the United States to assess 1-year mortality/hospitalization event rates per 100 patient-years (PYs), cumulative hospital health care costs per patient, and kidney-protective treatment use before/after SGLT-2i (dapagliflozin) approval for CKD (2021) for patients with CKD stage 3–4 with/without type 2 diabetes (T2D).

**Results:**

Among 449,232 patients (across-country median age range 74–81 years), 79% did not have T2D. Prevalence ranges for atherosclerotic cardiovascular disease and heart failure were 20%–36% and 17%–31%, respectively. Baseline kidney-protective treatment (renin–angiotensin system inhibitor and/or SGLT-2i) use was limited, especially among patients without T2D. Event rates were high for CKD (11.4–44.4/100 PYs) and heart failure (7.4–22.3/100 PYs). Up to 14.6% of patients had died within 1 year. Hospital costs were higher for CKD and heart failure than for atherosclerotic cardiovascular disease. After incident CKD, kidney-protective treatment initiation was low (8%–20%) and discontinuation was high (16%–27%), especially among patients without T2D.

**Conclusions:**

Incident CKD was associated with substantial morbidity, mortality, costs, and undertreatment, especially in patients without T2D, who represented the majority of patients. This highlights an urgent need for early CKD detection and better kidney-protective treatment use in moderate CKD.

## Visual Abstract

**Figure:**
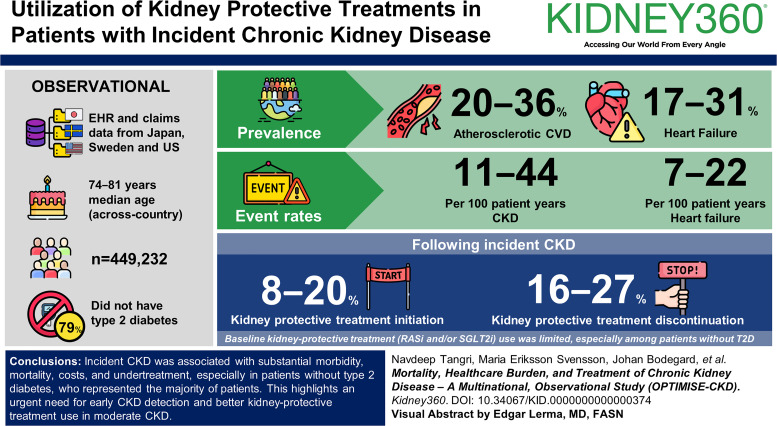

## Introduction

CKD is one of the most prevalent noncommunicable diseases globally, putting a huge burden on health care systems. CKD is estimated to affect more than 850 million people worldwide, and its prevalence is expected to increase with an aging population.^1–4^ For decades, treatment with renin–angiotensin system inhibitors (RASis) has been the mainstay of CKD treatment, with clinical trials showing kidney-protective effects and beneficial cardiovascular risk reduction.^5–8^

More recently, clinical trials have shown that sodium–glucose cotransporter-2 inhibitors (SGLT-2is) reduce the risk of CKD progression and cardiovascular events, regardless of diabetes status.^9^ Diabetes remains a leading cause of CKD in many countries; in the United States, more than 50% of patients with CKD have comorbid diabetes.^10^ Originally approved for use in type 2 diabetes (T2D), dapagliflozin was the first SGLT-2i approved for use in patients with CKD both with and without T2D. Both SGLT-2is and RASis are recommended as first-line CKD treatments in the 2023 Kidney Disease Improving Global Outcomes guidelines, regardless of diabetes status.^11^ Patients with CKD and without T2D are generally perceived as lower risk than those with T2D and may be less likely to be treated with disease-modifying therapy. However, patients without T2D may have other comorbidities, such as hypertension or cardiovascular disease, that require treatment to manage progression and the risk of adverse outcomes.^10^ Therefore, it is important to understand how novel and established kidney-protective treatments are used in real-world clinical practice in patients both with and without T2D.

This OPTIMISE-CKD study used contemporary real-world clinical data from well-established electronic health records and claims data sources from Japan, Sweden, and the United States. The aim was to describe the following: (*1*) clinical outcomes and hospital health care costs after incident CKD stage 3–4 to understand the urgency of prompt risk management and (*2*) how kidney-protective treatments (RASi and SGLT-2i) were used before and after the first SGLT-2i (dapagliflozin) was approved for the treatment of CKD.

## Methods

### Study Design

OPTIMISE-CKD is a multinational, observational, longitudinal cohort study that uses data extracted from electronic health records and claims data sources. The current analysis was performed using data from Japan, Sweden, and the United States (Supplemental Figure 1 and Supplemental Methods). Data sources used in this project are subject to ethical and privacy restrictions in each participating country (see Supplemental Methods for details).

### Study Populations and Study Periods

**Figure 1:**
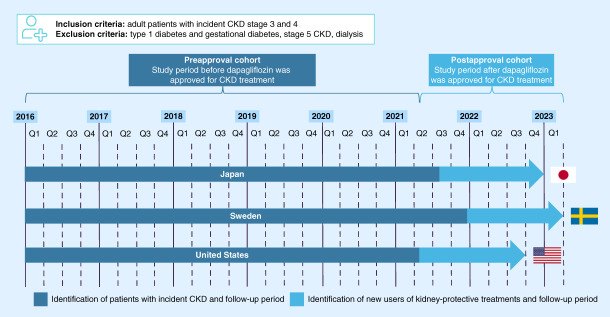
**Cohorts and study periods.** Index periods were January 1, 2016 to December 31, 2022 in Japan, January 1, 2016 to March 31, 2023 in Sweden, and January 1, 2016 to September 30, 2022 in the United States. Dapagliflozin was approved for CKD treatment in Japan on August 25, 2021, in Sweden on December 21, 2021, and in the United States on April 30, 2021. Q, quarter.

Patients aged 18 years or older were included if they met the CKD definition at any time during the study periods in each country (Figure 1 and Supplemental Table 1). CKD was defined as having either two eGFR measurements ≤60 ml/min per 1.73 m^2^ taken ≥90 days apart or a first eGFR ≤60 ml/min per 1.73 m^2^ followed by a first CKD diagnosis at any time, including chronic, acute, hypertensive, diabetic, tubular, and glomerular renal disease (Supplemental Table 2). Patients with CKD stage 5 (on the basis of eGFR <15 ml/min per 1.73 m^2^ or dialysis), dialysis, and type 1 or gestational diabetes were excluded. The overall study periods were January 1, 2016 to December 31, 2022 for Japan, January 1, 2016 to March 31, 2023 for Sweden, and January 1, 2016 to September 30, 2022 for the United States (Figure 1). For Sweden, two data sources were used to cover the entire study period (Supplemental Methods).

### Cohorts

Two cohorts were created for each country to study CKD during the periods before and after dapagliflozin was approved for the treatment of CKD (Figure 1; dapagliflozin approval dates: Japan, August 25, 2021; Sweden, December 21, 2021; the United States, April 30, 2021). In the preapproval cohort (2016–2021), patients were indexed at the date of incident CKD and followed up to the end of database, death, or loss to follow-up. In the postapproval cohort (2021–2023), substantially fewer patients and shorter follow-ups were expected if the same index were to be applied as for the preapproval cohort, and hence, patients with CKD and without T2D were indexed at the date of new initiation of kidney-protective treatments (RASi or SGLT-2i). New initiation was defined as first-ever use. For Sweden, a different data source was used for the postapproval cohort because the preapproval cohort data source did not cover the required period (Supplemental Methods).

### Patient Characteristics

For patients in both cohorts, characteristics were described before index (date of incident CKD), including demographics, comorbidities, and treatments (Supplemental Tables 2 and 3). Use of treatments was based on at least one filled prescription during the year before the index date (Supplemental Table 4). Patients with T2D were defined as those with recorded codes for glucose-lowering drugs and without diagnosis codes for type 1 diabetes or gestational diabetes. In Sweden, diagnosis codes were included for the preapproval cohort. Patients without T2D were defined as those without drug codes for glucose-lowering drugs and without diagnosis codes for type 1 diabetes or gestational diabetes (Supplemental Table 2).

### Clinical Outcomes

The following clinical outcomes were described for each patient in the preapproval cohort during the 12 months after the index date (incident CKD): inpatient hospitalizations with any diagnosis of CKD (including diagnoses of acute kidney failure, unspecified kidney failure, diabetic kidney disease, hypertensive CKD, dialysis, glomerular diseases, renal tubulointerstitial disease, or other), heart failure, myocardial infarction (MI), stroke, or peripheral artery disease (PAD) (Supplemental Table 2); all-cause hospitalization; and cardiovascular and all-cause mortality. Inpatient hospitalizations with a primary diagnosis of CKD, heart failure, MI, stroke, or PAD were also assessed.

### Hospital Health Care Costs

Within each country, charged costs for planned and unplanned inpatient and outpatient hospital visits associated with any of the following diagnoses were cumulatively summed for up to 5 years after the index date (date of incident CKD) for each patient in the preapproval cohort^1,12–14^: CKD, heart failure, MI, stroke, and PAD. The preapproval cohort was used for this analysis owing to the longer duration for which data were available. For this analysis, multiple diagnoses could be registered for a given hospitalization.

### Kidney-Protective Treatment Utilization

To study the effect of an incident CKD diagnosis on kidney-protective treatment use, RASi and/or SGLT-2i treatment status was assessed in the 3 months before and 12 months after incident CKD in the preapproval cohort. RASi/SGLT-2i treatment status during the 12 months after incident CKD was also assessed in patients in this cohort who were naive to RASi/SGLT-2i (defined as being treatment-naive for 12 months before initiation) or who were prevalent users of these treatments.

### RASi and Dapagliflozin Dose Utilization and Persistence

RASi persistence was assessed during the 12 months after index in patients with and without T2D in the preapproval cohort and in patients without T2D in the postapproval cohort. RASis were represented by several different types (11 ACE is and six angiotensin receptor blockers). Using the highest dose (HD) available in each country according to registered drug prescription doses, each RASi was categorized into three dose levels: low (<50% of the HD available), intermediate (50%–99% of the HD available), and high (100% of the HD available). This was performed separately within each country because the HD available for each RASi type varied across countries (Supplemental Table 5).

Dapagliflozin persistence was assessed during the 12 months after index in patients without T2D during the period after its approval for CKD in each country (postapproval cohort). Dapagliflozin persistence was assessed in patients without T2D to ensure that patients received dapagliflozin for the treatment of CKD and not for the T2D indication. Dapagliflozin treatment initiation was defined as the first-recorded dapagliflozin 10-mg prescription. Unlike RASis, which do not have a target dose for the treatment of CKD, dapagliflozin has a guideline-recommended target dose of 10 mg across all countries.^15^ It may also be used at a dose of 5 mg. Hence, dapagliflozin was categorized into two dose levels: target dose (10 mg; the approved dose for CKD treatment) and intermediate dose (5 mg).

For both RASis and dapagliflozin, the duration of each filled prescription was calculated based on the number of days covered by the number of pills contained in the box and the prescribed dose.^14,16–18^ Once a patient had used all the pills from a given prescription, the patient was considered to have discontinued treatment until a new prescription was filled.^14,18^ Hence, persistence is based on the proportion of patients on treatment at a given time point and is affected by both poor adherence and deliberate discontinuation.

### Statistical Analysis

Continuous variables were reported using medians and interquartile ranges. Categorical variables were reported as absolute frequencies (%). Proportions of patients using RASis or SGLT-2is after index were calculated as the actual number of patients with a prescription for these treatments covering each day divided by the total number of patients still in the database on that day. All analyses of event rates are descriptive, and no formal between-group comparisons were made. One-year event rates were calculated as events/100 patient-years (PYs) on the basis of time to first event.

The average costs related to each of the diagnoses were summarized as the arithmetic mean for each month, and the cumulative cost was the sum of the mean values from month 1 to the month of interest. For a patient to be included in the cost calculation for a given month, the follow-up period for the patient must cover the whole month (*e.g*., calculation of the costs for month 1 included only patients with at least 1 month of follow-up). Costs were extracted from data containing actual hospital visit costs, as charged by the health care provider, and included all first and repeated events associated with the targeted diagnoses (CKD, heart failure, MI, stroke, and PAD) during the follow-up. Overall costs for the hospital visit are reported; disease-specific costs are not reported. Both primary and secondary diagnoses were counted, and more than one of the targeted diagnoses may have contributed to the total cost of an individual hospital visit (*i.e*., there may have been multiple reasons recorded for a given hospitalization). Each diagnosis was analyzed independently from other diagnoses, and the hospital health care costs of two diagnoses could not be added to generate a combined cost. The results are presented separately for each country. There was no standardization or formal comparison made between countries.

A separate analysis of dapagliflozin uptake after its approval in each country was performed as follows. A separate CKD cohort was defined for each day from January 1, 2020, to the data extraction date. Patients were included if they had a CKD diagnosis before or at the date of dapagliflozin approval for CKD in each country. In the United States and Japan, patients also needed at least 6 months of available data before the date of interest. The proportion of these patients using dapagliflozin on a given date was calculated, where a patient was assumed to be using dapagliflozin if there was a dispense date before or at the date of interest, with a duration that lasted beyond that date.

## Results

### Baseline Characteristics (Preapproval Cohort)

In the preapproval cohort, in total, 449,232 patients with incident CKD stage 3–4 were identified in Japan (75,965 patients), Sweden (76,133 patients), and the United States (297,134 patients) (Table 1). Most patients did not have T2D (82%, 77%, and 78% non-T2D, in Japan, Sweden, and the United States, respectively). Across the three countries, the median age in the total population ranged from 74 to 81 years; the median age of patients with T2D was slightly lower than that of patients without T2D. There was some variation in the prevalence of heart failure and atherosclerotic cardiovascular disease (ASCVD) between the countries; in the total population, the prevalence of heart failure ranged from 17% to 31% and the prevalence of ASCVD ranged from 20% to 36%. The proportion of patients in the total population taking antihypertensive treatments (thiazides, RASi, and/or calcium channel blockers) at baseline in Japan, Sweden, and the United States was 31%, 68%, and 62%, respectively. In all countries, patients without T2D, who represented the majority of patients, were less treated with baseline kidney-protective, cardiovascular-protective, and antihypertensive treatments than those with T2D. For use of glucose-lowering treatments, see Supplemental Figure 2.

**Table 1:** Baseline characteristics

Variable	Japan			Sweden			The United States		
	Non-T2D	T2D	Total	Non-T2D	T2D	Total	Non-T2D	T2D	Total
Patients, *n* (%)	62,113 (82)	13,852 (18)	75,965 (100)	58,455 (77)	17,678 (23)	76,133 (100)	232,545 (78)	64,589 (22)	297,134 (100)
Age, yr, median (IQR)	82.0 (74.0–87.0)	78.0 (71.0–84.0)	81.0 (73.0–87.0)	78.3 (71.4–85.2)	76.5 (70.6–82.6)	77.8 (71.2–84.6)	75.0 (69.0–82.0)	72.0 (67.0–78.0)	74.0 (69.0–81.0)
Male, *n* (%)	32,633 (53)	8437 (61)	41,070 (54)	26,897 (46)	9527 (54)	36,424 (48)	83,917 (36)	25,888 (40)	109,805 (37)
**BMI, kg/m** ^**2**^ **, median (IQR)**	22.1 (19.4–24.8)	23.1 (20.5–26.0)	22.3 (19.6–25.0)	25.4 (22.5–28.7)	28.0 (24.7–31.8)	26.0 (23.0–29.6)	27.5 (24.1–31.6)	30.1 (26.3–34.8)	28.1 (24.5–32.4)
25.0 to <30 kg/m^2^, *n* (%)	8476 (19)	2739 (24)	11,215 (20)	9894 (34.0)	3699 (37.5)	13,593 (34.9)	20,226 (35)	5549 (32)	25,775 (34)
≥30.0 kg/m^2^, *n* (%)	1795 (4)	878 (8)	2673 (5)	5530 (19.0)	3481 (35.3)	9011 (23.1)	19,573 (34)	8782 (51)	28,355 (38)
**Comorbidities**									
ASCVD, *n* (%)	11,039 (18)	3823 (28)	14,862 (20)	18,248 (31)	6685 (38)	24,933 (33)	84,569 (36)	23,007 (36)	107,576 (36)
*Myocardial infarction*	3552 (6)	1515 (11)	5067 (7)	8388 (14)	3399 (19)	11,787 (15)	18,537 (8)	5848 (9)	24,385 (8)
*Stroke*	6189 (10)	1825 (13)	8014 (11)	10,054 (17)	3417 (19)	13,471 (18)	52,046 (22)	13,020 (20)	65,066 (22)
*Peripheral artery disease*	2446 (4)	1059 (8)	3505 (5)	2996 (5)	1309 (7)	4305 (6)	42,136 (18)	11,576 (18)	53,712 (18)
Atrial fibrillation/flutter, *n* (%)	10,210 (16)	2365 (17)	12,575 (17)	16,934 (29)	4573 (26)	21,507 (28)	40,147 (17)	8471 (13)	48,618 (16)
Heart failure, *n* (%)	18,693 (30)	5003 (36)	23,696 (31)	14,149 (24)	4103 (23)	18,252 (24)	38,412 (17)	11,266 (17)	49,678 (17)
CKD diagnosis, *n* (%)	23,355 (38)	4080 (29)	27,435 (36)	8560 (15)	2609 (15)	11,169 (15)	98,649 (42)	27,255 (42)	125,904 (42)
Cancer, *n* (%)	15,607 (25)	3781 (27)	19,388 (26)	20,253 (35)	5470 (31)	25,723 (34)	94,019 (40)	19,171 (30)	113,190 (38)
**Laboratory measurements^a^**									
Systolic BP, mm Hg, median (IQR)	N/A	N/A	N/A	136 (120–150)	135 (122–150)	136 (120–150)	129 (120–140)	130 (120–140)	130 (120–140)
*≥140 mm Hg,* n (%)	N/A	N/A	N/A	15,919 (47)	5338 (46)	21,257 (46)	4537 (26)	1446 (29)	5983 (27)
Diastolic BP, mm Hg, median (IQR)	N/A	N/A	N/A	78 (70–85)	75 (68–80)	76 (70–84)	N/A	N/A	N/A
*≥80 mm Hg,* n (%)	N/A	N/A	N/A	15,975 (47)	4736 (41)	20,711 (45)	N/A	N/A	N/A
Hemoglobin, g/dl, median (IQR)	11.5 (10.0–13.0)	11.6 (10.1–13.1)	11.5 (10.1–13.0)	13.2 (11.9–14.3)	13.0 (11.8–14.1)	13.1 (11.9–14.3)	13.2 (12.1–14.3)	12.8 (11.7–13.9)	13.2 (12.0–14.2)
Hematocrit, %, median (IQR)	35 (31–39)	35 (31–40)	35 (31–39)	40.0 (36.0–43.0)	39.0 (35.0–42.0)	40.0 (36.0–43.0)	40 (37–43)	39 (36–42)	40 (37–43)
Sodium, mmol/L, median (IQR)	141 (138–142)	140 (138–142)	140 (138–142)	140 (138–142)	139 (137–141)	140 (137–142)	141 (139–142)	140 (138–142)	140 (139–142)
Potassium, mmol/L, median (IQR)	4.3 (4.0–4.7)	4.4 (4.1–4.8)	4.4 (4.0–4.7)	4.2 (3.9–4.4)	4.2 (3.9–4.5)	4.2 (3.9–4.5)	4.4 (4.1–4.7)	4.5 (4.2–4.8)	4.4 (4.1–4.7)
*>5.5 mmol/L,* n (%)	5722 (9)	2271 (17)	7993 (11)	2503 (4.4)	1162 (6.7)	3665 (4.9)	11,232 (5)	4883 (8)	16,115 (5)
Magnesium, mmol/L, median (IQR)	2.1 (1.9–2.3)	2.1 (1.9–2.3)	2.1 (1.9–2.3)	N/A	N/A	N/A	2.0 (1.9–2.2)	1.9 (1.7–2.1)	2.0 (1.8–2.2)
Calcium, mmol/L, median (IQR)	8.9 (8.5–9.3)	8.9 (8.4–9.3)	8.9 (8.5–9.3)	N/A	N/A	N/A	9.5 (9.2–9.8)	9.6 (9.3–9.9)	9.5 (9.2–9.8)
HbA1c, mmol/mol (IFCC), median (IQR)	40 (37–44)	51 (44–60)	42 (38–49)	38.0 (36.0–41.0)	50.0 (44.0–59.0)	42.0 (37.0–49.0)	40 (37–43)	51 (44–61)	42 (38–49)
HbA1c, % (DCCT), median (IQR)	5.8 (5.5–6.2)	6.8 (6.2–7.6)	6.0 (5.6–6.6)	5.6 (5.4–5.9)	6.7 (6.2–7.5)	6.0 (5.5–6.6)	5.8 (5.5–6.1)	6.8 (6.2–7.7)	6.0 (5.6–6.6)
eGFR, ml/min per 1.73 m^2^, median (IQR)	49 (37–56)	51 (40–57)	49 (37–56)	53 (45–57)	52 (44–57)	53 (45–57)	53 (47–57)	53 (46–57)	53 (47–57)
*45–59 (stage 3a),* n (%)	37,072 (60)	9026 (65)	46,098 (61)	43,501 (74)	13,020 (74)	56,521 (74)	187,499 (81)	50,660 (78)	238,159 (80)
*30–44 (stage 3b),* n (%)	15,636 (25)	3131 (23)	18,767 (25)	10,889 (19)	3496 (20)	14,385 (19)	38,475 (17)	11,890 (18)	50,365 (17)
*15–29 (stage 4),* n (%)	9405 (15)	1695 (12)	11,100 (15)	4065 (7)	1162 (7)	5227 (7)	6571 (3)	2039 (3)	8610 (3)
Creatinine, mg/dl, median (IQR)	1.3 (1.0–1.5)	1.3 (1.1–1.5)	1.3 (1.0–1.5)	1.2 (1.0–1.4)	1.2 (1.1–1.4)	1.2 (1.0–1.4)	1.2 (1.1–1.4)	1.3 (1.1–1.4)	1.2 (1.1–1.4)
Serum albumin, g/dl, median (IQR)	3.6 (3.0–4.0)	3.6 (3.0–4.0)	3.6 (3.0–4.0)	N/A	N/A	N/A	N/A	N/A	N/A
UACR, mg/g, median (IQR)	N/A	N/A	N/A	13.3 (4.4–48.7)	15.9 (5.3–58.4)	14.2 (4.9–53.1)	8.3 (3.3–23.0)	12.4 (5.0–40.0)	10.0 (4.0–29.2)
*With UACR measurement,* n (%)	N/A	N/A	N/A	8173 (14)	6159 (35)	14,332 (19)	35,529 (15)	28,304 (44)	63,833 (21)
**Kidney-protective treatment, *n* (%)**									
RASi	15,597 (25)	7916 (57)	23,513 (31)	31,671 (54)	13,174 (75)	44,845 (59)	109,189 (47)	52,426 (81)	161,615 (54)
SGLT-2i	310 (<1)	1671 (12)	1981 (3)	12 (<1)	632 (4)	644 (1)	431 (<1)	4403 (7)	4834 (2)
Antihypertensive treatment,^b^ *n* (%)	15,715 (25)	7954 (57)	23,669 (31)	37,442 (64)	14,454 (82)	51,896 (68)	128,464 (55)	55,812 (86)	184,276 (62)
**ASCVD treatment, *n* (%)**									
Low-dose aspirin	6150 (10)	3423 (25)	9573 (13)	15,593 (27)	6530 (37)	22,123 (29)	48,360 (21)	18,101 (28)	66,461 (22)
Statins	8799 (14)	5618 (41)	14,417 (19)	19,209 (33)	10,910 (62)	30,119 (40)	97,800 (42)	49,275 (76)	147,075 (49)

Clinical and demographic characteristics of patients with incident CKD in Japan, Sweden, and the United States (preapproval cohort, *N*=449,232, 2016–2021). ASCVD, atherosclerotic cardiovascular disease; BMI, body mass index; DCCT, Diabetes Control and Complications Trial; HbA1c, glycated hemoglobin; IFCC, International Federation of Clinical Chemistry and Laboratory Medicine; IQR, interquartile range; N/A, not available or not applicable; RASi, renin–angiotensin system inhibitor; SGLT-2i, sodium–glucose cotransporter-2 inhibitor; T2D, type 2 diabetes; UACR, urine albumin-to-creatinine ratio.

a Laboratory measurements represent the last registered value in the year before incident CKD.

b Thiazides (low-ceiling diuretics), RASis, or calcium channel blockers (vasoactive/dihydropyridines).

### Clinical Outcomes (Preapproval Cohort)

**Figure 2:**
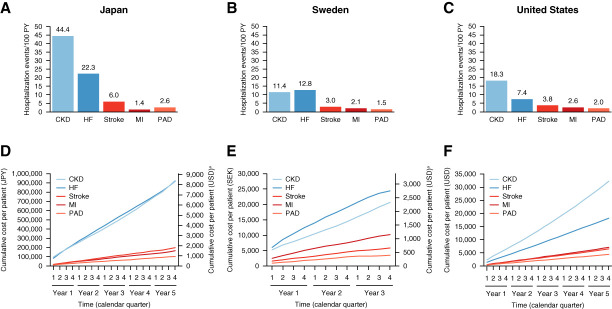
**Hospitalization risks and costs after incident CKD in 449,232 patients during the period before SGLT-2i approval for CKD (2016–2021).** (A–C) One-year event rates (events/100 PYs) for hospitalizations with any diagnosis of heart failure, CKD, MI, stroke, or PAD. (D–F) Average cumulative hospital health care costs per patient for hospitalizations associated with CKD, heart failure, MI, stroke, or PAD, reported in USD and local currency. ^a^Conversion rates from January 1, 2019 were used (1 JPY=0.0091 USD; 1 SEK=0.1127 USD); currency was converted simply by applying conversion rates, without considering differences in purchasing power. HF, heart failure; JPY, Japanese yen; MI, myocardial infarction; PAD, peripheral artery disease; PYs, patient years; SEK, Swedish krona; SGLT-2i, sodium–glucose cotransporter-2 inhibitor; USD, US dollars.

Risks of hospitalization with any diagnosis of CKD and/or heart failure were higher than for hospitalization with any diagnosis of ASCVD (MI, stroke, and PAD) across all countries (Figure 2, A–C). Similar rankings were also observed for hospitalizations with primary diagnoses of the five diseases (Supplemental Figure 3). All-cause hospitalization event rates were 93.5, 74.4, and 25.7 events/100 PYs in Japan, Sweden, and the United States, respectively (Supplemental Table 6). All-cause in- and out-of-hospital mortality was 14.6 events/100 PYs (data available for Sweden only). In-hospital mortality was 14.1, 8.7, and 6.5 events/100 PYs in Japan, Sweden, and the United States, respectively. Generally, fatal and nonfatal risks were either slightly higher or similar in patients without T2D versus with T2D.

### Hospital Health Care Costs (Preapproval Cohort)

Costs for CKD and/or heart failure hospitalizations after incident CKD were higher than those for ASCVD events across all countries (Figure 2, D–F). Hospitalization costs for patients with T2D were slightly higher than those for patients without T2D in all countries (Supplemental Figure 4).

### Kidney-Protective Treatment Use (Preapproval Cohort)

**Figure 3:**
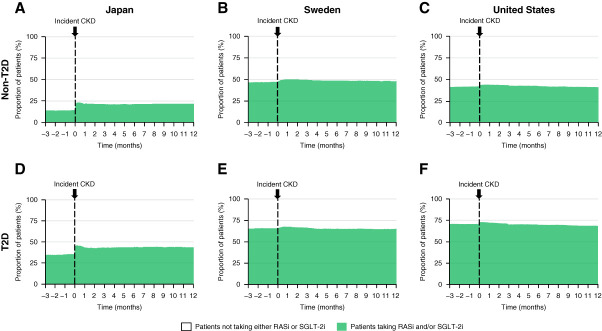
**Kidney-protective treatment use after incident CKD in 449,232 patients during the period before SGLT-2i approval for CKD (2016–2021).** Proportion of patients taking a RASi and/or SGLT-2i in the 3 months before and 12 months after incident CKD in patients (A–C) without T2D and (D–F) with T2D. RASi, renin–angiotensin system inhibitor; T2D, type 2 diabetes.

In the preapproval cohort, use of kidney-protective treatments (RASis and SGLT-2is) at index (date of incident CKD) was lower in patients without T2D (18%–48%) compared with those with T2D (43%–73%), and little change in use was observed in the 12 months after incident CKD in all countries (Figure 3). New initiation of kidney-protective treatments among patients naive to RASis and SGLT-2is in the preapproval cohort was 8%–20% at 12 months after incident CKD stage 3–4 (Supplemental Figure 5, A–C). Discontinuation among prevalent users was 16%–27% (Supplemental Figure 5, D–F).

**Figure 4:**
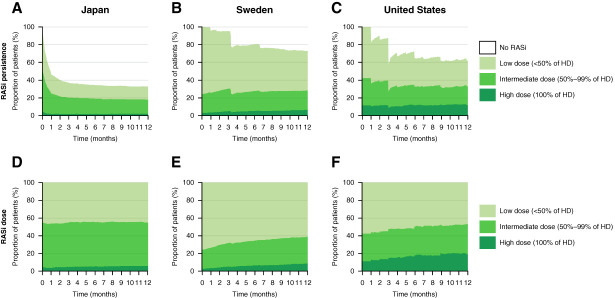
**RASi persistence and dose utilization in 449,232 patients during the period before SGLT-2i approval for CKD (2016–2021).** (A–C) RASi persistence and (D–F) dose use in patients with incident CKD after new RASi initiation. HD, highest dose.

Among patients initiated on RASis, persistence varied across countries: 33% of patients in Japan, 73% in Sweden, and 62% in the United States remained on treatment at 12 months after incident CKD (Figure 4, A–C). Of those patients remaining on RASi treatment, the majority were treated with low (45%–61%) or intermediate doses (30%–49%); high dose use was low (6%–19%) (Figure 4, D–F).

### Dapagliflozin and RASi Dose Utilization (Postapproval Cohort, Without T2D)

There were 115,443 patients in the postapproval cohort (Japan, 48,909; Sweden, 7168; United States, 59,366). Full baseline data for patients in the postapproval cohort are presented in Supplemental Table 7.

**Figure 5:**
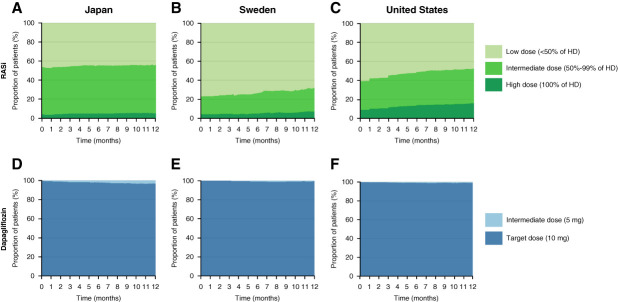
**RASi and dapagliflozin dose utilization in 115,443 patients during the period after first SGLT-2i (dapagliflozin) approval for CKD (2021–2023).** Proportion of patients with CKD and without T2D taking different doses of (A–C) RASi and (D–F) dapagliflozin after new RASi or dapagliflozin initiation.

Patients who were newly initiated on RASi in the pre- and postapproval cohorts had similar patterns of RASi persistence (Figure 5, A–C versus Figure 4, D–F) and dose utilization (Figure 4, A–C versus Supplemental Figure 6, A–C). Among patients in the postapproval cohort newly initiated on RASi, 79%, 25%, and 40% of patients in Japan, Sweden, and the United States, respectively, were not on RASi treatment 12 months after initiation (Supplemental Figure 6, A–C). Of those remaining on treatment, 5%, 7%, and 16% of patients in Japan, Sweden, and the United States, respectively, were treated with high doses (Figure 5, A–C).

Patients newly initiated on dapagliflozin showed varying levels of dapagliflozin persistence; 40%, 19%, and 49% of patients in Japan, Sweden, and the United States, respectively, were not on dapagliflozin treatment 12 months after initiation (Supplemental Figure 6, D–F). Of those remaining on treatment, 97%–99% were treated with the 10 mg target dose recommended for CKD, and very few patients received the 5 mg (intermediate) dose (Figure 5, D–F).

Dapagliflozin 10 mg uptake was low in the total population of patients with diagnosed CKD (3,690,290 patients; Japan, 1,210,675; Sweden, 193,302; United States, 2,286,313), especially among patients without T2D (Supplemental Figure 7). Overall, 1.4%, 5.1%, and 1.3% of patients were taking dapagliflozin 10 mg at the last available observation date (more than 12 months after approval) in Japan, Sweden, and the United States, respectively. Equivalent values for patients without T2D were 1.0%, 3.5%, and 0.3%, respectively.

## Discussion

In this large, contemporary study of patients with newly detected CKD stage 3–4 in Japan, Sweden, and the United States, we found that 79% of patients did not have T2D. Patients without T2D were less often treated with kidney- and cardiovascular-protective treatments at baseline than those with T2D. Even when disease-modifying therapies (RASi and/or SGLT-2i) were started, submaximal dosing and high discontinuation rates were common across all countries 1 year after initiation. Taken together, these findings highlight the important morbidity and mortality burden of CKD and the need for timely initiation and maintenance of kidney-protective treatments.

### Underutilization of Kidney-Protective Treatment

Treatment with a RASi and/or SGLT-2i provides many benefits to patients with CKD, including reducing albuminuria and proteinuria, delaying CKD progression, and preventing renal and cardiovascular events and death.^5–7,9^ The results of this study indicate substantial treatment inertia, that is, little increase in the use of kidney-protective treatments during the year after incident CKD stage 3–4 across all countries (Figure 3). The reasons for this treatment inertia might include low clinical awareness of CKD, underdiagnosis, and lack of knowledge concerning the benefits of RASi treatment in this patient population.^19^ The observed low 1-year initiation rates and substantial discontinuation rates of kidney-protective treatments were similar to results from a recent US study (1-year initiation 17.8%, discontinuation 56.0%) of patients with incident CKD that included only patients with T2D.^20^ The seriousness of discontinuation has been highlighted in a recent study that showed that stopping RASi treatment in CKD was associated with an increased risk of death and major cardiovascular events.^21,22^

The underutilization of preventive treatments observed in this study was especially pronounced in patients without T2D, who made up the majority of patients. This is despite these patients having similar risks of severe morbidity and mortality to patients with T2D. This may be partly because of the perceived lower risk of patients without T2D, the lower recommended follow-up frequency compared with patients with T2D, and differences in adherence to treatment recommendations and structured follow-up visits.

The low rate of RASi use in Japan may partly be explained by the fact that RASis are only indicated for hypertension in this country. In addition, Japanese CKD guidelines recommend caution when using RASis in elderly patients, and broader antihypertensive treatment strategies are applied in this country.^23^

The extensive use of low/intermediate RASi doses in this study is similar to what has previously been reported for the treatment of heart failure.^18,24^ RASis lack a target dose in the treatment of CKD, in contrast with heart failure for which RASi target doses exist. The effects of low/intermediate versus high RASi dose use in the treatment of CKD (and heart failure) are not fully understood and might affect the resulting risk control. Low RASi persistence and use of low/intermediate doses may be explained by initial eGFR decline, patients being older, or increased risk or perceived risk of side effects such as hyperkalemia, or hypotensive episodes.

### Postapproval Use of Kidney-Protective Treatments

RASi use changed little over time (little up-titration), and we did not observe substantial differences in dose use or discontinuation rates between the periods before and after dapagliflozin was approved for CKD. Most patients who remained on dapagliflozin 12 months after initiation maintained the 10 mg target dose.

The lowest rate of dapagliflozin persistence was in the United States. In contrast to Japan and Sweden, patients in the United States pay for their health care insurance and, depending on the insurance program, many also fully or partially pay for additional treatments. Patients in Japan and Sweden may also have to co-pay for treatment. However, in Sweden, an annual limit is applied to this co-payment, hence there are few/no financial consequences for the patient. This might explain the highest rate of dapagliflozin persistence in Sweden, where all drug treatment costs are covered by the public insurance system. These findings suggest that the effect of patient costs on access to treatment is an important modifiable factor in improving the use of these treatments. It is also important to note that treatment use was low in all countries after approval in this population, a finding similar to changes observed after heart failure indications.

### Mortality and Hospitalization Events and Costs

We noted that mortality in our study was higher in these patients with incident CKD stage 3–4 compared with 2.4 million patients with prevalent CKD in the CaReMe study, a contemporary study of patients with CKD stage 1–5 across 11 countries in Europe and Canada (including Sweden), in which 6%–9% of patients had died within a year.^1^ However, the patients in that study were slightly younger than in this study, and approximately a quarter had milder CKD (stage 1 or 2).^1^ Interestingly, across all countries, fatal and nonfatal risks were either slightly higher or similar in patients without T2D versus with T2D, contrary to the general perception of risk among these populations. Differences between patients with and without T2D may be explained partly by the slightly higher median age of patients without T2D versus with T2D. However, other factors could also explain the similar risks, for example, the fact that patients without T2D had fewer risk-lowering treatments and/or that the patients were selected after newly detected CKD. Nevertheless, this supports an increased awareness of risk in patients without T2D with newly detected CKD stage 3–4. Hospitalization rates and costs for cardiorenal complications (heart failure or CKD) were higher than for ASCVD across all countries and regardless of T2D status. This pattern is consistent with results from the CaReMe CKD study and contemporary studies of patients with heart failure and diabetes.^1,12–14,24^ A recent study also showed that patients with nondiabetic CKD are at high risk of serious adverse clinical outcomes, including worsening of CKD stage and hospitalization for heart failure.^25^ In combination with the results of the present study this study highlights that most patients with CKD, who do not have T2D, have medical needs that are currently not being met.

### Strengths and Limitations

Strengths of this study include the large size of the contemporaneous patient population, the multinational compilation of real-world health care data of total populations, and the consistency of the findings across the three countries, despite differences in demographics, treatment guidelines, and health care systems. Other strengths are the validity of the CKD definition used,^26,27^ the availability of a wide variety of patient clinical characteristics, and the availability of total hospital health care costs.

Limitations of this study include the limited generalizability to other countries with different health care systems or guidelines for the treatment of CKD and the limited follow-up available for patients in the postapproval cohort.

The Japan data set is limited to data from hospital settings only and therefore likely contains more patients with more advanced disease progression/comorbidities than the Sweden and United States databases in which patients were additionally identified in general practice. As Japan and the United States lack nationwide death registries, full coverage data for all-cause mortality (both inside and outside of hospital) were available only for Sweden. Given that Swedish patients had lower and higher all-cause hospitalization risks compared with Japan and the United States, respectively, the observed country-specific overall risk profiles might suggest that total death risks are higher in Japan and lower in the United States compared with Sweden. This is further supported by the in-hospital death risk being higher in Japan and lower in the United States than in Sweden.

Reasons for prescriptions were not recorded; hence, patients may have received kidney-protective treatments for indications other than CKD (*e.g*., RASis for hypertension). Doses used during follow-up may have been under-/overestimated if any dose changes were not adequately recorded. Patients who were defined as having discontinued treatment were allowed to restart treatment during the 12 months of follow-up. Hence, our analysis may have overestimated discontinuation and underestimated target/high dose achievement for these treatments.

In conclusion, in Japan, Sweden, and the United States, most patients with incident CKD stage 3–4 did not have T2D. After incident CKD stage 3–4, adverse outcomes were common, mainly driven by hospitalizations for CKD and heart failure and death, regardless of T2D status. A large proportion of patients were not receiving guideline-recommended treatment with RASis and/or SGLT-2is, especially those without T2D, highlighting a need for improved risk management and treatment initiation in this high-risk group of patients. Although dapagliflozin uptake was low/slow after its approval for CKD, patients treated with dapagliflozin had a high likelihood of maintaining the 10 mg target dose. Overall, these results highlight an urgent need for early CKD detection and better kidney-protective treatment use to improve patient outcomes, regardless of T2D status.

## Supplementary Material

## Data Availability

Data cannot be shared. Data were sourced from proprietary databases.

## Supplemental Material

Supplemental Methods

Supplemental Table 1. Inclusion and exclusion criteria.

Supplemental Table 2. Definitions of CKD and comorbidities.

Supplemental Table 3. Baseline definitions.

Supplemental Table 4. Definitions of drug treatments.

Supplemental Table 5. Categorization of different types of RASi and doses.

Supplemental Table 6. Hospitalization and mortality risks in patients with incident CKD.

Supplemental Table 7. Baseline characteristics of new users of dapagliflozin and RASi.

Supplemental Figure 1. Description of data sources.

Supplemental Figure 2. Glucose-lowering drug utilization in patients with type 2 diabetes.

Supplemental Figure 3. Hospitalization risks after incident CKD.

Supplemental Figure 4. Costs after incident CKD stratified by T2D status.

Supplemental Figure 5. Kidney-protective treatment use among naive and prevalent users.

Supplemental Figure 6. RASi and dapagliflozin persistence and dose utilization.

Supplemental Figure 7. Total dapagliflozin use before and after its approval for CKD.

Supplemental Video. Mortality, Health Care Burden, and Treatment of CKD: A Multinational, Observational Study (OPTIMISE-CKD).

## References

[1] Sundstrom J Bodegard J Bollmann A, .; CaReMe CKD Investigators. Prevalence, outcomes, and cost of chronic kidney disease in a contemporary population of 2·4 million patients from 11 countries: the CaReMe CKD study. Lancet Reg Health Eur. 2022;20:100438. doi:10.1016/j.lanepe.2022.100438 36090671 PMC9459126

[2] Hill NR Fatoba ST Oke JL, . Global prevalence of chronic kidney disease - a systematic review and meta-analysis. PLoS One. 2016;11(7):e0158765. doi:10.1371/journal.pone.0158765 27383068 PMC4934905

[3] GBD Chronic Kidney Disease Collaboration. Global, regional, and national burden of chronic kidney disease, 1990-2017: a systematic analysis for the Global Burden of Disease Study 2017. Lancet. 2020;395(10225):709–733. doi:10.1016/S0140-6736(20)30045-3 32061315 PMC7049905

[4] Jager KJ Kovesdy C Langham R Rosenberg M Jha V Zoccali C. A single number for advocacy and communication–worldwide more than 850 million individuals have kidney diseases. Nephrol Dial Transplant. 2019;34(11):1803–1805. doi:10.1093/ndt/gfz174 31566230

[5] Lewis EJ Hunsicker LG Bain RP Rohde RD. The effect of angiotensin-converting-enzyme inhibition on diabetic nephropathy. The Collaborative Study Group. N Engl J Med. 1993;329(20):1456–1462. doi:10.1056/NEJM199311113292004 8413456

[6] Lewis EJ Hunsicker LG Clarke WR, .; Collaborative Study Group. Renoprotective effect of the angiotensin-receptor antagonist irbesartan in patients with nephropathy due to type 2 diabetes. N Engl J Med. 2001;345(12):851–860. doi:10.1056/NEJMoa011303 11565517

[7] Brenner BM Cooper ME de Zeeuw D, .; RENAAL Study Investigators. Effects of losartan on renal and cardiovascular outcomes in patients with type 2 diabetes and nephropathy. N Engl J Med. 2001;345(12):861–869. doi:10.1056/NEJMoa011161 11565518

[8] Xie X Liu Y Perkovic V, . Renin-angiotensin system inhibitors and kidney and cardiovascular outcomes in patients with CKD: a bayesian network meta-analysis of randomized clinical trials. Am J Kidney Dis. 2016;67(5):728–741. doi:10.1053/j.ajkd.2015.10.011 26597926

[9] Nuffield Department of Population Health Renal Studies Group, SGLT2 inhibitor Meta-Analysis Cardio-Renal Trialists' Consortium. Impact of diabetes on the effects of sodium glucose co-transporter-2 inhibitors on kidney outcomes: collaborative meta-analysis of large placebo-controlled trials. Lancet. 2022;400(10365):1788–1801. doi:10.1016/S0140-6736(22)02074-8 36351458 PMC7613836

[10] United States Renal Data System. 2022 USRDS Annual Data Report: Epidemiology of Kidney Disease in the United States. National Institutes of Health, National Institute of Diabetes and Digestive and Kidney Diseases, Bethesda, MD, 2022.

[11] Kidney Disease Improving Global Outcomes. KDIGO 2023 CKD Guideline: A Brave New World on the Evaluation, Prognostication, and Management of Kidney Disease @ ERA 2023. Accessed June 28, 2023. https://kdigo.org/conferences/era-2023-ckd-guideline-draft-preview/

[12] Norhammar A Bodegard J Eriksson JW, .; CaReMe Cardiorenal Investigators. Cost of healthcare utilization associated with incident cardiovascular and renal disease in individuals with type 2 diabetes: a multinational, observational study across 12 countries. Diabetes Obes Metab. 2022;24(7):1277–1287. doi:10.1111/dom.14698 35322567 PMC9321691

[13] Norhammar A Bodegard J Vanderheyden M, . Prevalence, outcomes and costs of a contemporary, multinational population with heart failure. Heart. 2023;109(7):548–556. doi:10.1136/heartjnl-2022-321702 36781285 PMC10086499

[14] Bozkurt B Savarese G Adamsson Eryd S, . Mortality, outcomes, costs, and use of medicines following a first heart failure hospitalization: EVOLUTION HF. JACC Heart Fail. 2023;11(10):1320–1332. doi:10.1016/j.jchf.2023.04.017 37354145

[15] Navaneethan SD Zoungas S Caramori ML, . Diabetes management in chronic kidney disease: synopsis of the KDIGO 2022 clinical practice guideline update. Ann Intern Med. 2023;176(3):381–387. doi:10.7326/M22-2904 36623286

[16] Angeras O Hasvold P Thuresson M Deleskog A ÖBraun O. Treatment pattern of contemporary dual antiplatelet therapies after acute coronary syndrome: a Swedish nationwide population-based cohort study. Scand Cardiovasc J. 2016;50(2):99–107. doi:10.3109/14017431.2015.1119304 26564402 PMC4819834

[17] Halvorsen S Jortveit J Hasvold P Thuresson M Øie E. Initiation of and long-term adherence to secondary preventive drugs after acute myocardial infarction. BMC Cardiovasc Disord. 2016;16:115. doi:10.1186/s12872-016-0283-6 27246583 PMC4886431

[18] Savarese G Bodegard J Norhammar A, . Heart failure drug titration, discontinuation, mortality and heart failure hospitalization risk: a multinational observational study (US, UK and Sweden). Eur J Heart Fail. 2021;23(9):1499–1511. doi:10.1002/ejhf.2271 34132001

[19] Tangri N Moriyama T Schneider MP, . Prevalence of undiagnosed stage 3 chronic kidney disease in France, Germany, Italy, Japan and the USA: results from the multinational observational REVEAL-CKD study. BMJ Open. 2023;13(5):e067386. doi:10.1136/bmjopen-2022-067386 PMC10230905 37217263

[20] Fried L Schmedt N Folkerts K, . High unmet treatment needs in patients with chronic kidney disease and type 2 diabetes: real-world evidence from a US claims database. Nephrol Dial Transplant. 2023;38(3):630–643. doi:10.1093/ndt/gfac140 35389468 PMC9976755

[21] Fu EL Evans M Clase CM, . Stopping renin-angiotensin system inhibitors in patients with advanced CKD and risk of adverse outcomes: a nationwide study. J Am Soc Nephrol. 2021;32(2):424–435. doi:10.1681/ASN.2020050682 33372009 PMC8054897

[22] Tang C Wen XY Lv J, . Discontinuation of renin-angiotensin system inhibitors and clinical outcomes in chronic kidney disease: a systemic review and meta-analysis. Am J Nephrol. 2023;54(5-6):234–244. doi:10.1159/000531000 37231791 PMC10614243

[23] Japanese Society of Nephrology. Essential points from evidence-based clinical practice guidelines for chronic kidney disease 2018. Clin Exp Nephrol. 2019;23(1):1–15. doi:10.1007/s10157-018-1648-1 30506489 PMC6344397

[24] Savarese G Kishi T Vardeny O, . Heart failure drug treatment – inertia, titration, and discontinuation: a multinational observational study (EVOLUTION HF). JACC Heart Fail. 2023;11(1):1–14. doi:10.1016/j.jchf.2022.08.009 36202739

[25] Wanner C Schuchhardt J Bauer C, . Clinical characteristics and disease outcomes in non-diabetic chronic kidney disease: retrospective analysis of a US healthcare claims database. J Nephrol. 2023;36(1):45–54. doi:10.1007/s40620-022-01340-x 35567698 PMC9895008

[26] Birkeland KI Bodegard J Banerjee A, . Lower cardiorenal risk with sodium-glucose cotransporter-2 inhibitors versus dipeptidyl peptidase-4 inhibitors in patients with type 2 diabetes without cardiovascular and renal diseases: a large multinational observational study. Diabetes Obes Metab. 2021;23(1):75–85. doi:10.1111/dom.14189 32893440 PMC7756303

[27] Birkeland KI Bodegard J Eriksson JW, . Heart failure and chronic kidney disease manifestation and mortality risk associations in type 2 diabetes: a large multinational cohort study. Diabetes Obes Metab. 2020;22(9):1607–1618. doi:10.1111/dom.14074 32363737 PMC7496468

