# Supplementary material for: Mortality, Health Care Burden, and Treatment of CKD: A Multinational, Observational Study (OPTIMISE-CKD)
Source: Kidney360. 2024 Feb 1;5(3):352–62. doi: 10.34067/KID.0000000000000374 (PMC11000732; doi:10.34067/KID.0000000000000374)
Supplement: Supplementary file 1 [file kidney360-5-352-s001.docx]

**SUPPLEMENTAL MATERIAL**

Mortality, Healthcare Burden, and Treatment of Chronic Kidney Disease – a Multinational, Observational study (OPTIMISE-CKD)

Tangri, Navdeep;^1^ Svensson, Maria K;^2^ Bodegård, Johan;^3^ Adamsson Eryd, Samuel;^3^ Thuresson, Marcus;^4^ Gustafsson, Stefan;^5^ Sofue, Tadashi^6^

1. University of Manitoba Max Rady College of Medicine, Winnipeg, MB, Canada
2. Department of Medical Sciences, Renal Medicine, Uppsala University, Uppsala, Sweden
3. Cardiovascular, Renal and Metabolism Evidence, BioPharmaceuticals Medical, AstraZeneca, Gothenburg, Sweden
4. Statisticon AB, Uppsala, Sweden
5. Sence Research AB, Uppsala, Sweden
6. Department of Cardiorenal and Cerebrovascular Medicine, Kagawa University, Kagawa, Japan

**Contents**

[Supplemental Methods 4](#_Toc152848757)

[Data Sources 4](#_Toc152848758)

[Supplemental Table 1: Inclusion and Exclusion Criteria. 7](#_Toc152848759)

[Supplemental Table 2: Definitions of CKD and Comorbidities. 8](#_Toc152848760)

[Supplemental Table 3: Baseline Definitions. 9](#_Toc152848761)

[Supplemental Table 4: Definitions of Drug Treatments. 10](#_Toc152848762)

[Supplemental Table 5: Categorization of Different Types of RASi and Doses. 11](#_Toc152848763)

[Supplemental Table 6: Hospitalization and Mortality Risks in Patients with Incident CKD. 12](#_Toc152848764)

[Supplemental Table 7: Baseline Characteristics of New Users of Dapagliflozin and RASi 13](#_Toc152848765)

[Supplemental Figure 1: Description of Data Sources. 15](#_Toc152848766)

[Supplemental Figure 2: Glucose-Lowering Drug Utilization in Patients with Type 2 Diabetes 16](#_Toc152848767)

[Supplemental Figure 3: Hospitalization Risks Following Incident CKD. 17](#_Toc152848768)

[Supplemental Figure 4: Costs Following Incident CKD Stratified by T2D Status. 18](#_Toc152848769)

[Supplemental Figure 5: Kidney-protective Treatment Use Among Naive and Prevalent Users. 19](#_Toc152848770)

[Supplemental Figure 6: RASi and Dapagliflozin Persistence and Dose Utilization. 20](#_Toc152848771)

[Supplemental Figure 7: Total Dapagliflozin Use Before and After its Approval for CKD. 21](#_Toc152848772)

[References 22](#_Toc152848773)

# Supplemental Methods

## Data Sources

The study uses secondary data extracted from well-established electronic health records or claims data sources in Japan, Sweden, and the United States. A short description of each data source is provided below. For Sweden, different data sources were used for the pre-approval and post-approval cohorts because the pre-approval cohort data source did not cover the required time period.

***Japan***

Residents in Japan (Japanese or non-Japanese) are all covered by mandatory and universal national healthcare coverage.^1^ We used the Medical Data Vision (MDV) database, a hospital-based database containing administrative claims and laboratory data, linked to the Diagnostic Procedure Combination (flat-fee payment system) inpatient hospital payment system, covering 39 million patients across Japan.^2-4^ Demographic characteristics, including age and sex distributions of these patients, are similar to those of available statistics in Japan.^5-8^ The dataset includes information on patients’ disease diagnoses, blood test results from approximately 10% of the patient population, dosage and administration period of treatments, and all medical practice such as dates of surgery and types of examinations. A previous report has examined the coverage of dispensed drugs, comparing MDV and JMDC (a receipt database that includes data on both inpatients and outpatients and medical check-ups obtained from multiple employee-based health insurance societies) data sources, suggesting that coverage of drug utilization data may not be an issue.^9^

In Japan, ethical approval and informed consent do not apply to the use of de-identified secondary data according to the Japanese Ethical Guidelines for Medical and Health Research Involving Human Subjects.

***Sweden***

*Laboratory Detailed Data Source (Pre-Approval Cohort)*

For the pre-approval cohort, the CELOSIA chronic kidney disease (CKD) study database was used. This database includes patient information from three linked national Swedish registries with virtually complete coverage of the Swedish population: the Prescribed Drug Register (2005–2021) covering all filled treatment prescriptions using the Anatomical Therapeutic Chemical (ATC) codes; the Cause of Death Registry (1961–2021); and the National Patient Registry covering all open patient clinic visit diagnoses for the years 2001–2021 and all hospital discharge diagnoses for the years 1987–2021.^10^ Diagnoses are recorded according to the International Classification of Diseases (ICD) system and recording has been shown to be of high validity.^11^ All three national registers are held by the Swedish National Board of Health and Welfare (NBHW). Regional data on laboratory and clinical measurements from electronic health records (EHRs) in Region Stockholm (2.4 million inhabitants; 24% of the Swedish population) and Region Skåne (1.34 million inhabitants; 13% of the Swedish population) are also included. The data linkage of the study database was performed by NBHW using unique personal identification numbers.^12^

The CELOSIA CKD study population comprises all patients in Sweden with at least one diagnosis of CKD, kidney disease, or diabetes mellitus recorded in the National Patient Register during the time period January 1, 2000 to January 1, 2019 or all patients in Region Stockholm and Region Skåne with at least one of the following laboratory test results recorded in EHRs within the public healthcare during the time period of 2010 (2013 for Region Skåne) to January 1, 2019: B-type natriuretic peptide (BNP) (>100 ng/L), N-terminal pro-B-type natriuretic peptide (NT-proBNP) (>300 ng/L), estimated glomerular filtration rate (eGFR) (<60 mL/min), glycated hemoglobin (HbA1c) (>48 mmol/L), urine albumin-to-creatinine ratio (UACR) >3 mg/mmoL.

For the study population, the CELOSIA CKD dataset covers information on age, sex, diagnoses (ICD10), clinical procedure codes, and treatment utilization for all patients in Sweden, as well as clinical measurements (such as height, weight, body mass index [BMI], blood pressure, smoking habits) and results from laboratory tests for patients in Region Stockholm and Region Skåne.

The study was approved by the Ethical Review Authority (reference number 2020-03850). The CELOSIA dataset was separately managed by Sence Research AB (Uppsala, Sweden).

*Nationwide Data Source* (*Post-Approval Cohort*)

The study database includes patient information from three linked national Swedish registries with full coverage of the Swedish population: the Prescribed Drug Register (July 2005 to January 2023), covering all filled treatment prescriptions using the ATC codes; the Cause of Death Registry (1987–2023); and the National Patient Registry covering all open patient clinic visit diagnoses for 2001–2023 and all hospital discharge diagnoses for the years 1987–2023.^13^ Diagnoses are recorded according to the ICD system and recording has been shown to be of high validity.^11^ All three registers are held by the NBHW, who also performed the data linkage using unique personal identification numbers.^12^

The study was approved by the Stockholm Regional Ethics Committee (reference numbers 2020-05714 and 2013/2206-31), with data linkage performed by the Swedish National Board of Health and Welfare. The linked database was separately managed by Statisticon AB (Uppsala, Sweden).

***United States***

Optum’s de-identified Clinformatics® Data Mart (CDM) is an administrative claims database for a privately insured population in the United States with commercial or Medicare Advantage coverage. The database covers more than 78 million people with data collected since January 2007 and contains patient-level data from claims submitted for all medical and pharmacy health care services with information related to health care costs and resource utilization. The population is geographically diverse, spanning all 50 states.

For the hospital healthcare cost analysis, the data source provided only charged cost, which is an unreliable measure of healthcare expenditure in the United States, and we were not able to use actual costs reimbursed by payers (plan paid amounts).

As this was an analysis of de-identified claims data compliant with guidance related to the Health Insurance Portability and Accountability Act (HIPAA) privacy rule,^14^ institutional review board (IRB) approval was not required. As per Title 45 of the Code of Federal Regulations (CFR), Part 46,^15^ the administrative claims data analysis in this study was exempt from IRB review because it was a retrospective analysis of existing data (hence no patient intervention or interaction), and no patient-identifiable information was included in the claims dataset.

Supplemental Table 1: Inclusion and Exclusion Criteria.

| **Inclusion Criteria** |
| --- |
| - Age ≥18 years as of study index date - With first-ever registered laboratory-confirmed CKD or CKD diagnosis, defined as having either of the following:   - two eGFR measurements ≤60 mL/min/1.73m^2^ taken ≥90 days apart **or**   - or a first eGFR measurement ≤60 mL/min/1.73 m^2^ followed by a first CKD diagnosis^a^ |
| **Exclusion Criteria** |
| - History of stage 5 CKD, dialysis, type 1 diabetes, or gestational diabetes on or before index date |

^a^A full list of diagnosis codes used to identify CKD can be found in Supplemental Table 2.

CKD, chronic kidney disease; eGFR, estimated glomerular filtration rate.

Supplemental Table 2: Definitions of CKD and Comorbidities.

| **Disease** | **ICD-10** | **Surgical Code/Drug Treatment** |
| --- | --- | --- |
| Heart failure | I50, I11.0, I13.0, I13.2 |  |
| CKD | N17-N19, I12.0-I12.9, I13.1, I13.2, N00-N08, N10-N16 N08.3, E10.2, E11.2, E12.2, E13.2, E14.2, Z49, Z99.2 | Codes for dialysis |
| Type 2 diabetes | Excluding: E10 (type 1 diabetes), O24.4 (gestational diabetes) | Codes for glucose-lowering drugs: A10 |
| No type 2 diabetes | Excluding: E10 (type 1 diabetes), O24.4 (gestational diabetes) | No codes for glucose-lowering drugs: A10 |
| Type 1 diabetes | E10 |  |
| Cardiovascular disease |  |  |
| Myocardial infarction | I21-I22, I25.2, I25.6 |  |
| Ischemic heart disease | I21-I22, I25.2, I25.6, I20.9, I20.0 | Codes for revascularizations |
| CABG/PCI |  | Codes for revascularizations |
| Unstable angina | I20.0 |  |
| Angina pectoris | I20.1, I20.8, I20.9, I25.1, I25.5 | Nitrates: C01DA |
| Atrial fibrillation | I48 |  |
| Stroke | I60-I66, G45 |  |
| Hemorrhagic | I60-I62 |  |
| Ischemic | I63 |  |
| Transitory ischemic attack | G45 |  |
| Peripheral artery disease | I70.2, I73.9, I74.2-9 | Code for limb angioplasty |
| Device therapy: Cardioverter and pacemaker |  | Code for device implantation |
| Other disease |  |  |
| Hyperkalemia | E875 |  |
| Cancer | C00-C99 |  |
| COPD | J44 |  |
| COVID-19 infection | U07.1, U07.2 |  |

CABG, coronary artery bypass graft; CKD, chronic kidney disease; COPD, chronic obstructive pulmonary disease; COVID-19, coronavirus disease 2019; ICD, International Classification of Diseases; PCI, percutaneous coronary intervention.

Supplemental Table 3: Baseline Definitions.

| **Variable** | **Definition** |
| --- | --- |
| Sex | Male or female |
| Age | Age at index date |
| Index year/month | Year/month of index date |

Supplemental Table 4: Definitions of Drug Treatments.

| **Treatment Category/**  **Class/Medicine** | **ATC Code** | **ATC WHO Code** |
| --- | --- | --- |
| RASi | ACE or ARB or ARNI |  |
| ACEi | C09A, C09B | C09A, C09B |
| ARB | C09C, C09D (excluding C09DX04) | C09C, C09D excluding treatment name ENTRESTO |
| SGLT-2i | A10BK, A10BD09, A10BD11, A10BD12, A10BD15, A10BD19, A10BD20, A10BD21, A10BD23, A10BD24 | A10P |
| Dapagliflozin | A10BK01, A10BD15, A10BD21 | A10P + treatment name: FORXIGA |
| Antihypertensive treatments |  |  |
| RASi | ACE or ARB |  |
| Calcium channel blockers | C08C |  |
| Thiazides | C03A |  |
| Other treatments |  |  |
| Statins | C10AA | C10A1 |
| Low-dose acetylic salicylic acid | B01AC06, B01AC56, C10BX01, C10BX02, C10BX04, C10BX05, C10BX06, C10BX08, C10BX12, C07FX02, C07FX03, C07FX04, B01AC86, B01AC36, B01AC34 | B01C1 |

ACEi, angiotensin-converting enzyme inhibitor; ARB, angiotensin receptor blocker; ARNI, angiotensin receptor-neprilysin inhibitor; ATC, Anatomical Therapeutic Chemical; RASi, renin–angiotensin system inhibitor; SGLT-2i, sodium–glucose cotransporter-2 inhibitor; WHO, World Health Organization

Supplemental Table 5: Categorization of Different Types of RASi and Doses Available in Japan, Sweden, and the United States.

| **RASi** | **Japan** | | | **Sweden** | | | **United States** | | |
| --- | --- | --- | --- | --- | --- | --- | --- | --- | --- |
|  | **Low**  **(mg)** | **Intermediate (mg)** | **High**  **(mg)** | **Low**  **(mg)** | **Intermediate (mg)** | **High**  **(mg)** | **Low**  **(mg)** | **Intermediate (mg)** | **High**  **(mg)** |
| **ACEi** |  |  |  |  |  |  |  |  |  |
| Alacepril | 12.5 | 25 | 50 | N/A | N/A | N/A | N/A | N/A | N/A |
| Benazepril | N/A | N/A | N/A | N/A | N/A | N/A | 5–10 | 20 | 40 |
| Captopril | N/A | 12.5 | 25 | N/A | 25 | 50 | 12.5 | 25 | 50 |
| Enalapril | 2.5 | 5 | 10 | 2.5–5 | 10 | 20 | 2.5–5 | 10 | 20 |
| Fosinopril | N/A | N/A | N/A | N/A | N/A | N/A | 10 | 20 | 40 |
| Imidapril | 2.5 | 5 | 10 | N/A | N/A | N/A | N/A | N/A | N/A |
| Lisinopril | 5 | 10 | 20 | 5 | 10 | 20 | 2.5–5 | 20–30 | 40 |
| Perindopril | N/A | 2 | 4 | N/A | N/A | N/A | 2 | 4 | 8 |
| Quinapril | N/A | N/A | N/A | N/A | N/A | N/A | 5–10 | 20 | 40 |
| Ramipril | N/A | N/A | N/A | 1.25–2.5 | 10 | 20 | 1.25–2.5 | 5 | 10 |
| Trandolapril | N/A | N/A | N/A | N/A | N/A | N/A | 1 | 2 | 4 |
| **ARB** |  |  |  |  |  |  |  |  |  |
| Candesartan | 2–4 | 8 | 12 | 4–8 | 16 | 32 | 4–8 | 16 | 32 |
| Irbesartan | 50 | 100 | 200 | 75 | 150 | 300 | 75 | 150 | 300 |
| Losartan | 25 | 50 | 100 | 12.5–25 | 50 | 100 | 25 | 50 | 100 |
| Olmesartan | 10 | 20 | 40 | N/A | N/A | N/A | 5 | 20 | 40 |
| Telmisartan | 20 | 40 | 80 | 20 | 40 | 80 | 20 | 40 | 80 |
| Valsartan | 20–40 | 80 | 160 | 40–80 | 160 | 320 | 40–80 | 160 | 320 |

ACEi, angiotensin-converting enzyme inhibitor; ARB, angiotensin receptor blocker; N/A, not available; RASi, renin–angiotensin system inhibitor.

Supplemental Table 6: Hospitalization and Mortality Risks in Patients with Incident CKD. One-year event rates (events per 100 patient-years) for hospitalizations with any diagnosis of CKD, HF, myocardial infarction, stroke or peripheral artery disease, all-cause hospitalization, and mortality among patients with incident CKD in Japan, Sweden, and the USA stratified by T2D status at index and overall (pre-approval cohort, N=449 232, 2016–2021).

| **Event** | **Events per 100 Patient-Years (N events)^a^** | | | | | | | | |
| --- | --- | --- | --- | --- | --- | --- | --- | --- | --- |
|  | **Japan** | | | **Sweden** | | | **USA** | | |
|  | **Non-T2D**  n=62 113 | **T2D**  n=13 852 | **Overall**  n=75 965 | **Non-T2D**  n=58 455 | **T2D**  n=17 678 | **Overall**  n=76 133 | **Non-T2D**  n=232 545 | **T2D**  n=64 589 | **Overall**  n=297 134 |
| **Cardiorenal complications** |  |  |  |  |  |  |  |  |  |
| CKD | 47.0 (16 652) | 34.2 (3074) | 44.4 (19 726) | 11.1 (5228) | 12.3 (1730) | 11.4 (6958) | 18.2 (34 937) | 18.7 (10 008) | 18.3 (44 945) |
| HF | 23.0 (9011) | 19.7 (1890) | 22.3 (10 901) | 13.0 (6085) | 12.3 (1728) | 12.8 (7813) | 7.4 (15 035) | 7.7 (4370) | 7.4 (19 405) |
| **ASCVD** |  |  |  |  |  |  |  |  |  |
| Myocardial infarction | 1.4 (582) | 1.5 (156) | 1.4 (738) | 2.0 (993) | 2.5 (372) | 2.1 (1365) | 2.6 (5368) | 2.8 (1651) | 2.6 (7019) |
| Stroke | 6.1 (2566) | 5.3 (543) | 6.0 (3109) | 2.9 (1414) | 3.3 (484) | 3.0 (1898) | 3.7 (7743) | 4.0 (2315) | 3.8 (10 058) |
| Peripheral artery disease | 2.4 (1005) | 3.4 (347) | 2.6 (1352) | 1.4 (672) | 2.1 (306) | 1.5 (978) | 2.0 (4136) | 1.9 (1099) | 2.0 (5235) |
| **All-cause hospitalization** | 97.3 (29 718) | 78.3 (6091) | 93.5 (35 809) | 74.3 (25 720) | 74.9 (7730) | 74.4 (33 450) | 25.9 (48 200) | 24.9 (13 005) | 25.7 (61 205) |
| **Mortality** |  |  |  |  |  |  |  |  |  |
| All-cause death (in hospital) | 14.1 (6055) | 14.1 (1476) | 14.1 (7531) | 8.8 (4386) | 8.4 (1261) | 8.7 (5647) | 6.8 (14 448) | 5.4 (3211) | 6.5 (17 659) |
| All-cause death (in and out of hospital) | N/A^b^ | N/A^b^ | N/A^b^ | 14.7 (7363) | 14.2 (2116) | 14.6 (9479) | N/A^b^ | N/A^b^ | N/A^b^ |
| Cardiovascular death | N/A^c^ | N/A^c^ | N/A^c^ | 4.7 (2352) | 4.3 (635) | 4.6 (2987) | N/A^c^ | N/A^c^ | N/A^c^ |

^a^Based on first event during the 12 months following index.

^b^Data on deaths outside of hospital not available.

^c^Cause of death registries not available.

ASCVD, atherosclerotic cardiovascular disease; CKD, chronic kidney disease; HF, heart failure; N/A, not available; T2D, type 2 diabetes.

Supplemental Table 7: Baseline Characteristics of New Users of Dapagliflozin and RASi Without T2D in Japan, Sweden, and the United States (post-approval cohort, N=115 443, 2021–2023).

| **Variable** | **Japan** | | **Sweden** | | **USA** | |
| --- | --- | --- | --- | --- | --- | --- |
|  | Dapagliflozin  10 mg | RASi | Dapagliflozin  10 mg | RASi | Dapagliflozin  10 mg | RASi |
| Number of patients, n (%) | 10 959 (22) | 37 950 (78) | 3464 (48) | 3704 (52) | 6643 (11) | 52 723 (89) |
| Age, years, median (IQR) | 75.0 (63.0–85.0) | 78.0 (69.0–86.0) | 76.0 (63.0–83.0) | 74.0 (57.0–83.0) | 74.0 (69.0–80.0) | 72.0 (65.0–80.0) |
| Male, n (%) | 7479 (68) | 23 251 (61) | 2372 (68) | 2260 (61) | 3884 (58) | 26 796 (51) |
| **Comorbidities** |  |  |  |  |  |  |
| ASCVD, n (%) | 2876 (26) | 8373 (22) | 824 (24) | 698 (19) | 4025 (61) | 21 090 (40) |
| Myocardial infarction, n (%) | 1254 (11) | 2816 (7) | 653 (19) | 462 (12) | 2080 (31) | 6971 (13) |
| Stroke, n (%) | 1426 (13) | 4517 (12) | 149 (4) | 214 (6) | 2186 (33) | 11 840 (22) |
| Peripheral artery disease, n (%) | 824 (8) | 2061 (5) | 111 (3) | 109 (3) | 2146 (32) | 10 152 (19) |
| Atrial fibrillation/flutter, n (%) | 2865 (26) | 5738 (15) | 1533 (44) | 958 (26) | 2737 (41) | 9358 (18) |
| HF, n (%) | 5842 (53) | 14 132 (37) | 2000 (58) | 1042 (28) | 4111 (62) | 11 356 (22) |
| CKD diagnosis, n (%) | 10 959 (100) | 37 950 (100) | 3464 (100) | 3704 (100) | 6643 (100) | 52 723 (100) |
| Cancer, n (%) | 1744 (16) | 7239 (19) | 446 (13) | 510 (14) | 2566 (39) | 17 739 (34) |
| **Laboratory measurements^a^** |  |  |  |  |  |  |
| Systolic BP, mmHg, median (IQR) | N/A | N/A | N/A | N/A | 128 (116–139) | 138 (126–150) |
| ≥140 mmHg, n (%) | N/A | N/A | N/A | N/A | 849 (24) | 9137 (46) |
| Diastolic BP, mmHg, median (IQR) | N/A | N/A | N/A | N/A | N/A | N/A |
| ≥80 mmHg, n (%) | N/A | N/A | N/A | N/A | N/A | N/A |
| Hemoglobin, g/dL, median (IQR) | 12.5 (11.1–14.2) | 11.6 (10.1–13.3) | N/A | N/A | 13.1 (11.7–14.3) | 13.7 (12.5–14.9) |
| Hematocrit, %, median (IQR) | 38 (34–43) | 36 (31–40) | N/A | N/A | 40 (36–43) | 41 (38–45) |
| Sodium, mmol/L, median (IQR) | 140 (139–142) | 140 (138–142) | N/A | N/A | 140 (138–142) | 140 (138–142) |
| Potassium, mmol/L, median (IQR) | 4.3 (4.1–4.7) | 4.2 (3.9–4.6) | N/A | N/A | 4.4 (4.1–4.7) | 4.3 (4.1–4.6) |
| >5.5 mmol/L, n (%) | 234 (14) | 753 (11) | N/A | N/A | 539 (11) | 1345 (4) |
| Magnesium, mmol/L, median (IQR) | 2.1 (1.9–2.3) | 2.0 (1.9–2.2) | N/A | N/A | 2.0 (1.9–2.2) | 2.0 (1.9–2.2) |
| Calcium, mmol/L, median (IQR) | 9.1 (8.7–9.4) | 8.9 (8.4–9.3) | N/A | N/A | 9.4 (9.1–9.7) | 9.4 (9.2–9.7) |
| eGFR, mL/min/1.73 m^2^, median (IQR) | 44 (31–62) | 54 (30–77) | N/A | N/A | 48 (38–61) | 62 (50–78) |
| 45–59 (Stage 3a), n (%) | 361 (22) | 1133 (16) | N/A | N/A | 1559 (31) | 9776 (29) |
| 30–44 (Stage 3b), n (%) | 458 (28) | 1123 (16) | N/A | N/A | 1627 (32) | 4668 (14) |
| 15–29 (Stage 4), n (%) | 328 (20) | 907 (13) | N/A | N/A | 497 (10) | 1037 (3) |
| Creatinine, mg/dL, median (IQR) | 1.4 (1.1–1.9) | 1.2 (0.9–1.9) | N/A | N/A | 1.3 (1.1–1.6) | 1.0 (0.9–1.2) |
| Serum albumin, g/dL, median (IQR) | 3.8 (3.4–4.1) | 3.5 (2.9–3.9) | N/A | N/A | N/A | N/A |
| UACR, mg/g, median (IQR) | N/A | N/A | N/A | N/A | 28.0 (9.0–163.5) | 16.0 (7.0–59.0) |
| With UACR measurement, n (%) | N/A | N/A | N/A | N/A | 1720 (26) | 7650 (15) |
| **Kidney-protective treatment** |  |  |  |  |  |  |
| RASi, n (%) | 5302 (48) | 0 (0) | 2913 (84) | 0 (0) | 3944 (59) | 0 (0) |
| SGLT-2i, n (%) | 0 (0) | 817 (2) | 0 (0) | 360 (10) | 0 (0) | 529 (1) |
| **Antihypertensive treatment,^b^ n (%)** | N/A | N/A | N/A | N/A | N/A | N/A |
| **ASCVD treatment** |  |  |  |  |  |  |
| Low-dose aspirin, n (%) | 1050 (10) | 2034 (5) | 843 (24) | 882 (24) | 1203 (18) | 10 475 (20) |
| Statins, n (%) | 3307 (30) | 2961 (8) | 1957 (56) | 1469 (40) | 4571 (69) | 20 910 (40) |

^a^Laboratory measurements represent the last registered value in the year prior to the date of new initiation of dapagliflozin or RASi.

^b^Thiazides (low-ceiling diuretics), RASis, or calcium channel blockers (vasoactive/dihydropyridines).

ASCVD, atherosclerotic cardiovascular disease; BMI, body mass index; BP, blood pressure; CKD, chronic kidney disease; eGFR, estimated glomerular filtration rate; HF, heart failure; IQR, interquartile range; N/A, not available or not applicable; RASi, renin–angiotensin system inhibitor; SGLT-2i, sodium–glucose co‑transporter‑2 inhibitor; T2D, type 2 diabetes; UACR, urine albumin-to-creatinine ratio.

Supplemental Figure 1: Description of Data Sources. Color coding indicates where data were available and were used for this study (green=data available and used; orange=data not available). Data extractions are based on the following levels of healthcare: 1, primary healthcare; 2, secondary healthcare (specialist or outpatient hospital care); 3, tertiary healthcare (in-hospital care).


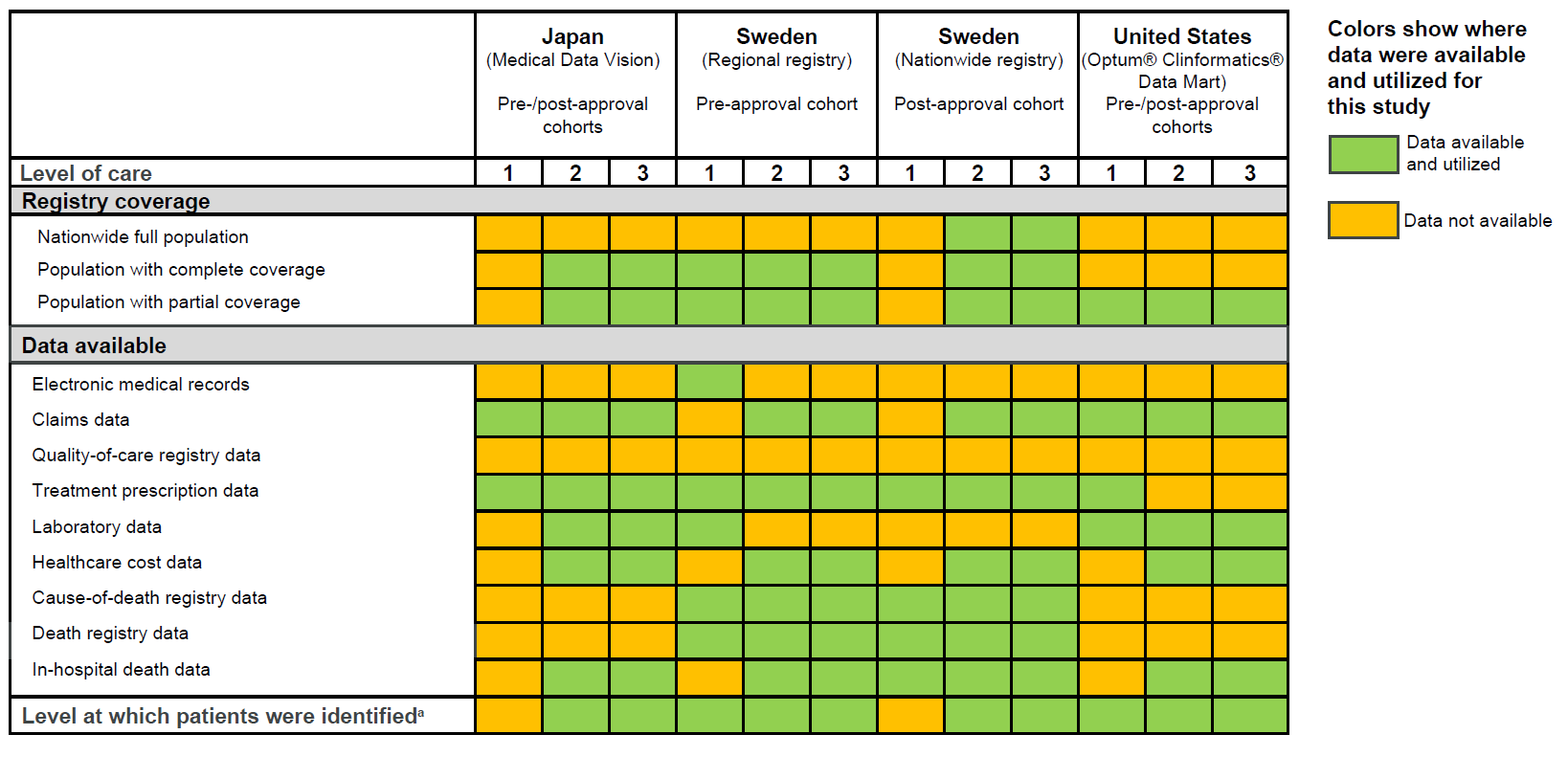


^a^Describing the level of care (primary, secondary, or tertiary) at which patients were indexed for the study, ie level at which incident CKD was identified or new kidney-protective treatments were initiated.

Supplemental Figure 2: Glucose-Lowering Drug Utilization in Patients with Type 2 Diabetes in 449 232 Patients in Japan, Sweden and the United States During the Period Before SGLT2i Approval for CKD (2016–2021).


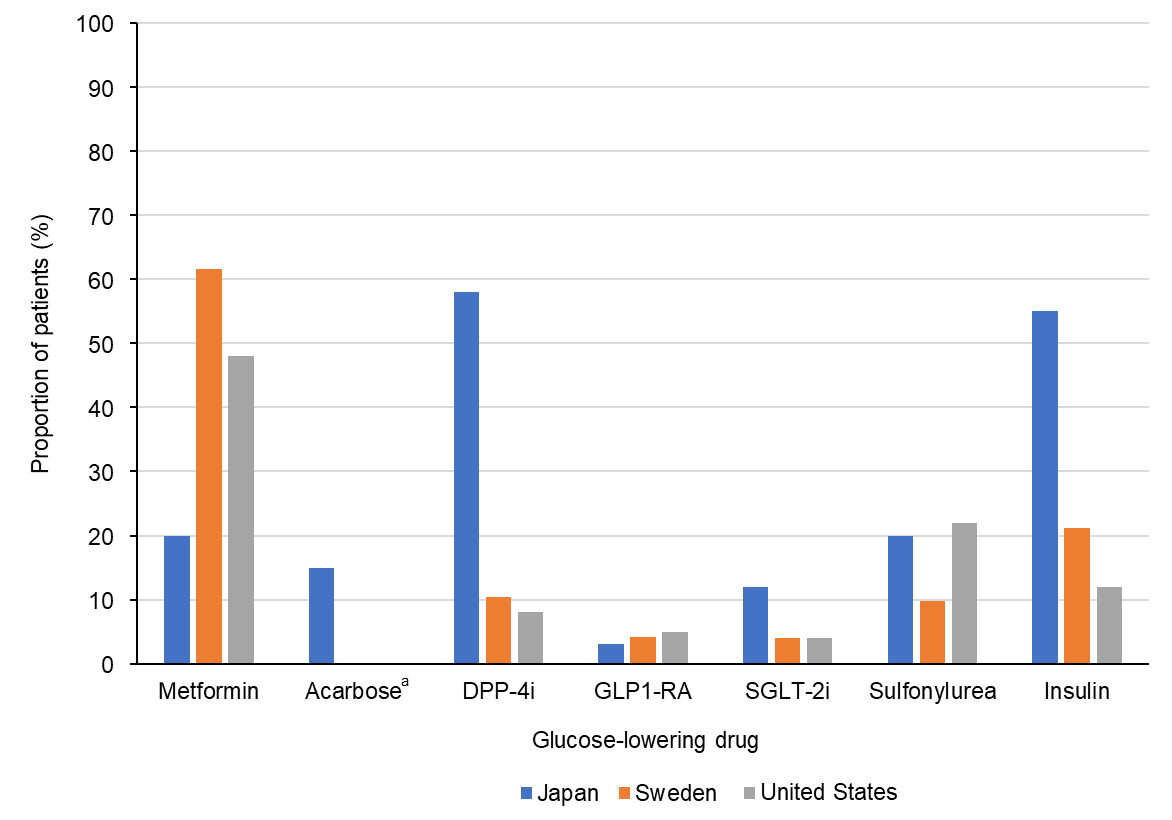


^a^Data on acarbose use in Japan was included because acarbose is often used instead of metformin in Asian countries.

DPP-4i, dipeptidyl peptidase 4 inhibitor; GLP1-RA, glucagon-like peptide-1 receptor agonist; SGLT-2i, sodium–glucose co‑transporter‑2 inhibitor.

Supplemental Figure 3: Hospitalization Risks Following Incident CKD in 449 232 Patients During the Period Before SGLT2i Approval for CKD (2016–2021). One-year event rates (events/100 PY) for hospitalizations with a primary diagnosis of HF, CKD, MI, stroke, or PAD.


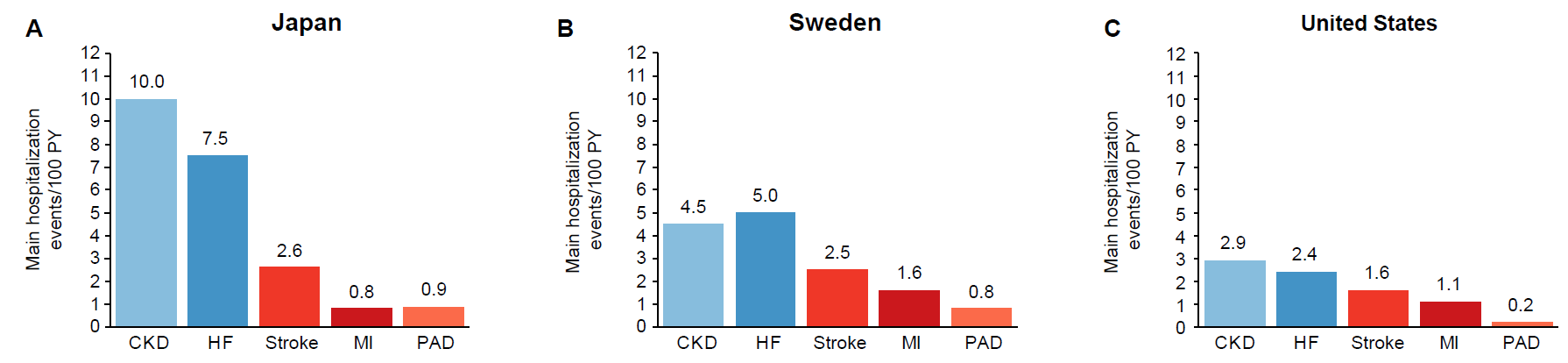


CKD, chronic kidney disease; HF, heart failure; MI, myocardial infarction; PAD, peripheral artery disease; PY, patient year; SGLT-2i, sodium–glucose co‑transporter‑2 inhibitor.

Supplemental Figure 4: Costs Following Incident CKD Stratified by T2D Status in 449 232 Patients During the Period Before SGLT2i Approval for CKD (2016–2021). Average cumulative hospital healthcare costs per patient for hospitalizations associated with CKD, HF, MI, stroke, or PAD among patients (A–C) without T2D and (D–F) with T2D, reported in USD and local currency.


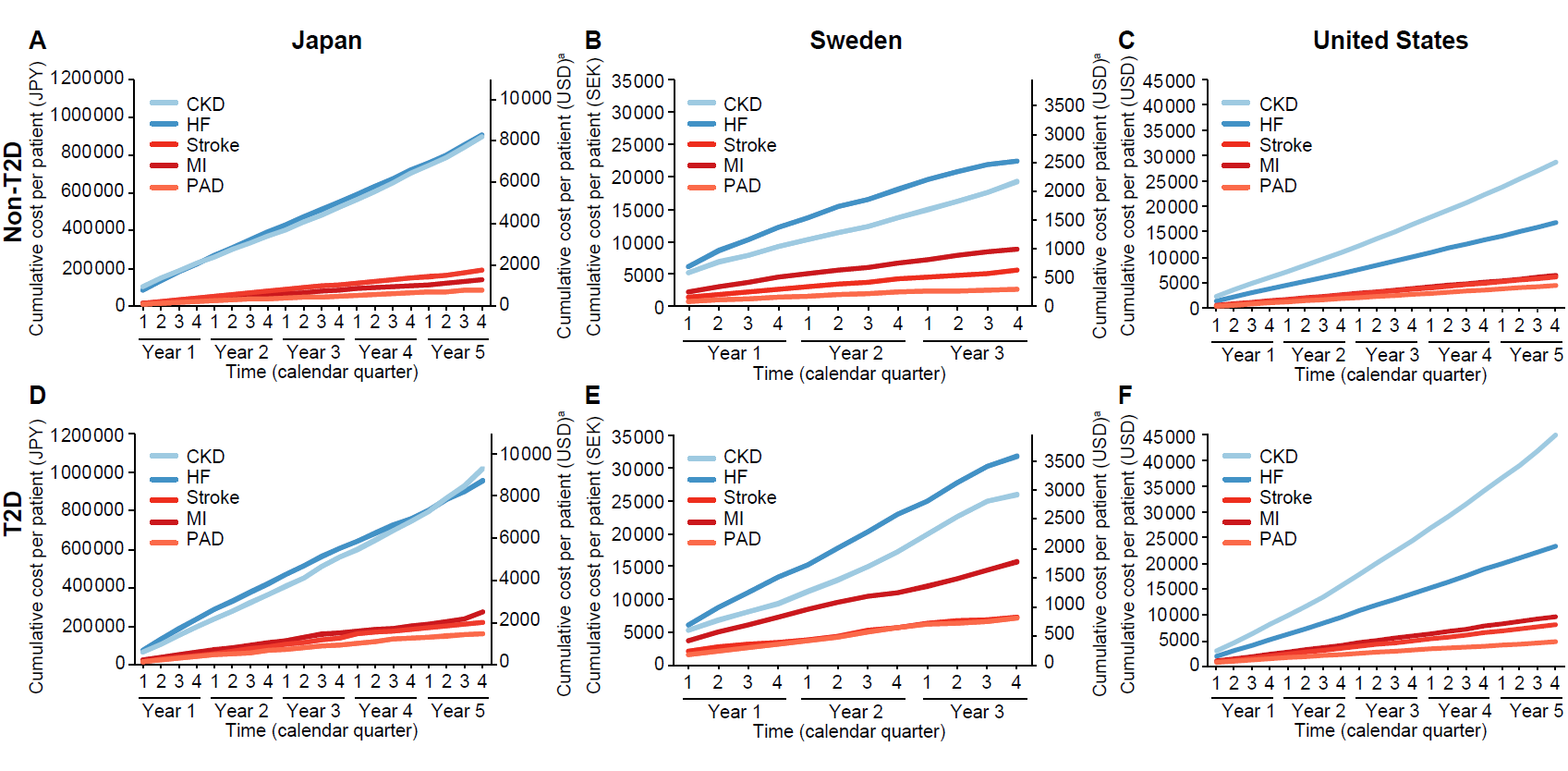


^a^Conversion rates from January 1, 2019 were used (1 JPY=0.0091 USD; 1 SEK=0.1127 USD); currency was converted simply by applying conversion rates, without considering differences in purchasing power.

CKD, chronic kidney disease; HF, heart failure; JPY, Japanese yen; MI, myocardial infarction; PAD, peripheral artery disease; SEK, Swedish krona; SGLT-2i, sodium–glucose co‑transporter‑2 inhibitor; T2D, type 2 diabetes; USD, US dollars.

Supplemental Figure 5: Kidney-protective Treatment Use Among Naive and Prevalent Users Following Incident CKD in 449 232 Patients During the Period Before SGLT2i Approval for CKD (2016–2021). Proportions of patients taking and not taking either a RASi or SGLT-2i, among patients with incident CKD, with and without T2D, who were (A–C) naive to RASi/SGLT-2i or (D–F) prevalent users of these treatments.


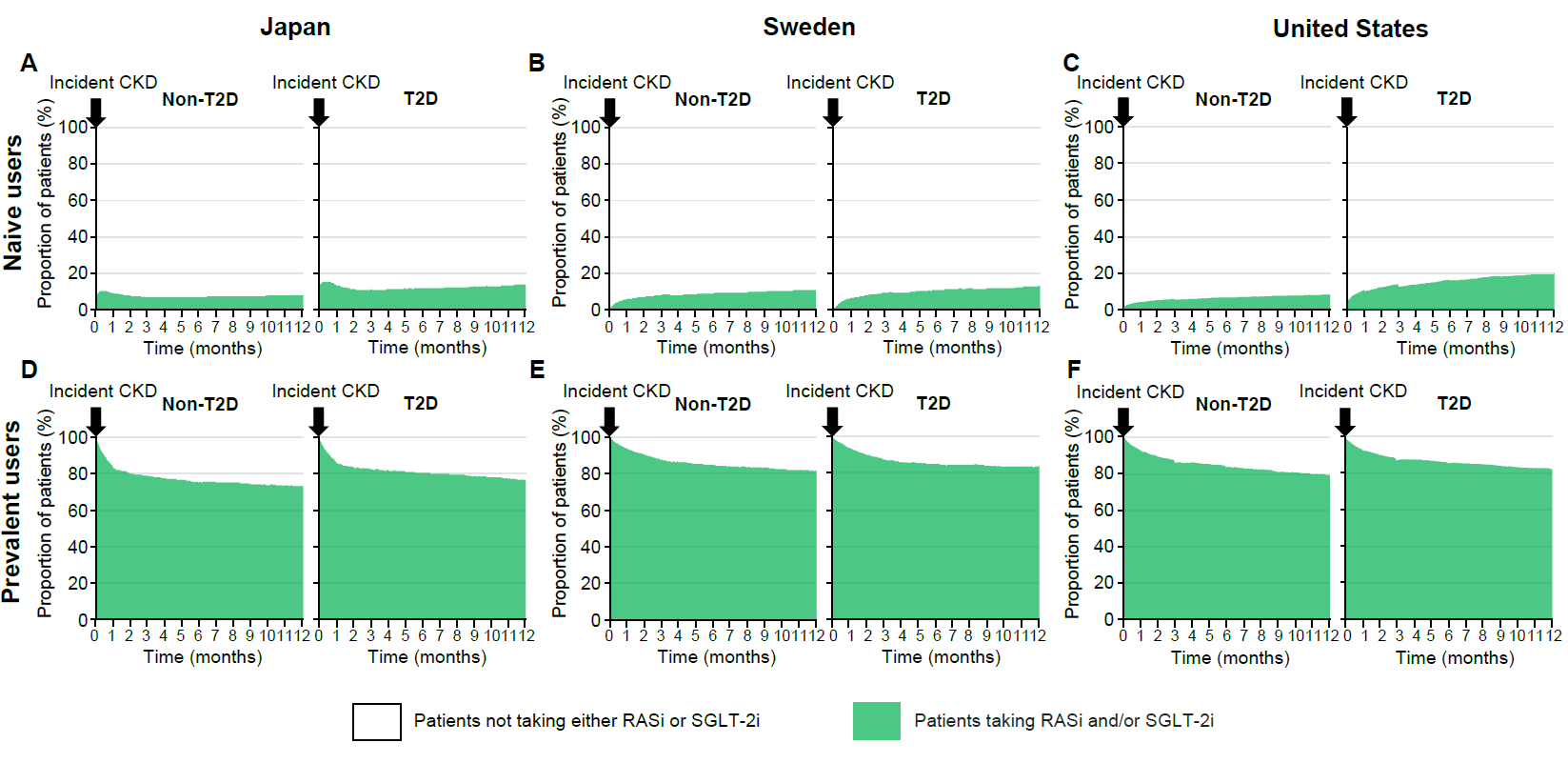


CKD, chronic kidney disease; HD, highest dose; RASi, renin–angiotensin system inhibitor; SGLT-2i, sodium–glucose cotransporter-2 inhibitor; T2D, type 2 diabetes.

Supplemental Figure 6: RASi and Dapagliflozin Persistence and Dose Utilization in 115 443 Patients During the Period after First SGLT2i (dapagliflozin) Approval for CKD (2021–2023). Proportion of patients with CKD and without T2D not taking and taking different doses of (A–C) RASi and (D–F) dapagliflozin following new RASi or dapagliflozin initiation.


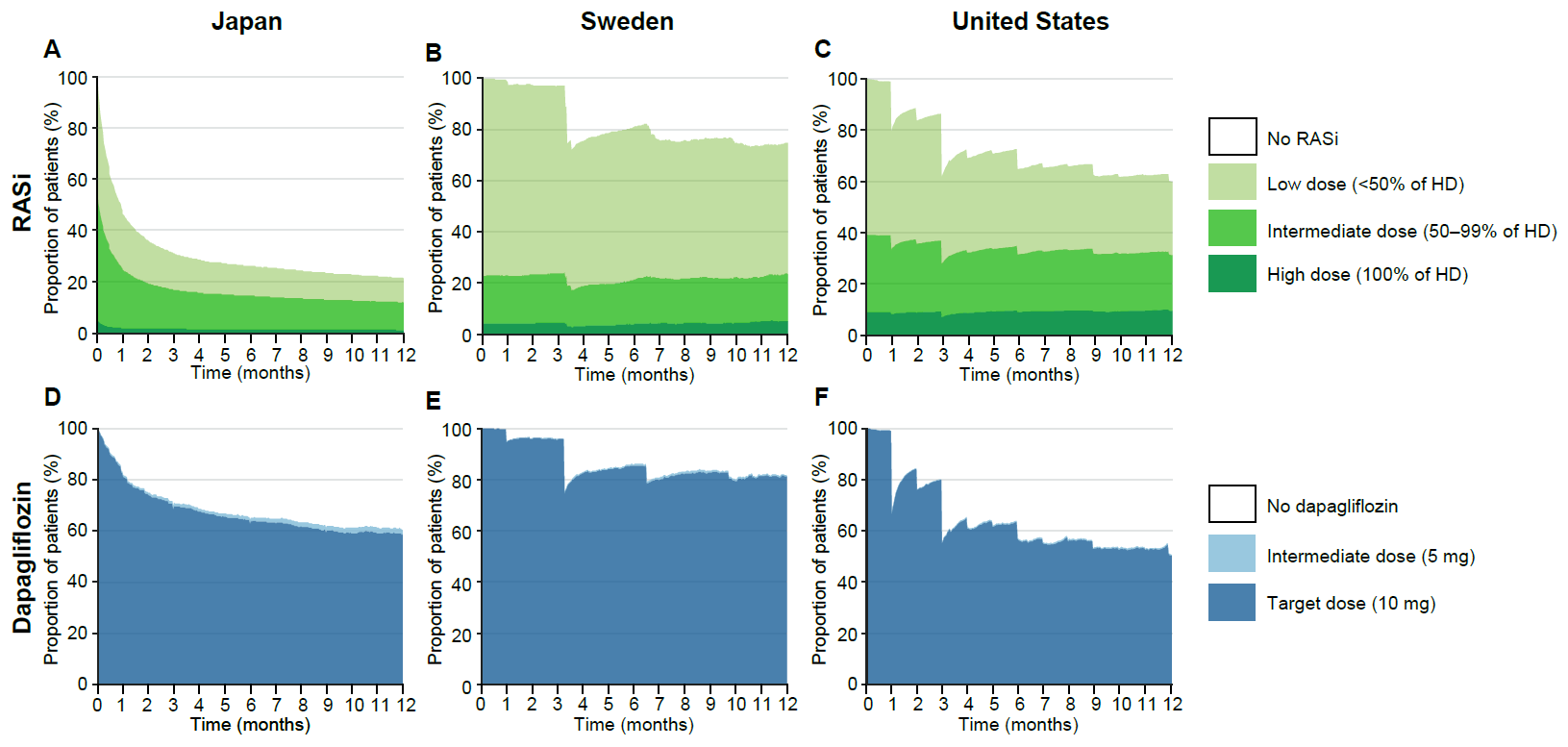


CKD, chronic kidney disease; HD, highest dose; RASi, renin–angiotensin system inhibitor; SGLT-2i, sodium–glucose cotransporter-2 inhibitor; T2D, type 2 diabetes.

Supplemental Figure 7: Total Dapagliflozin Use Before and After its Approval for CKD. Proportion of all patients treated with dapagliflozin 10 mg with a CKD diagnosis, (A–C) without and (D–F) with T2D.


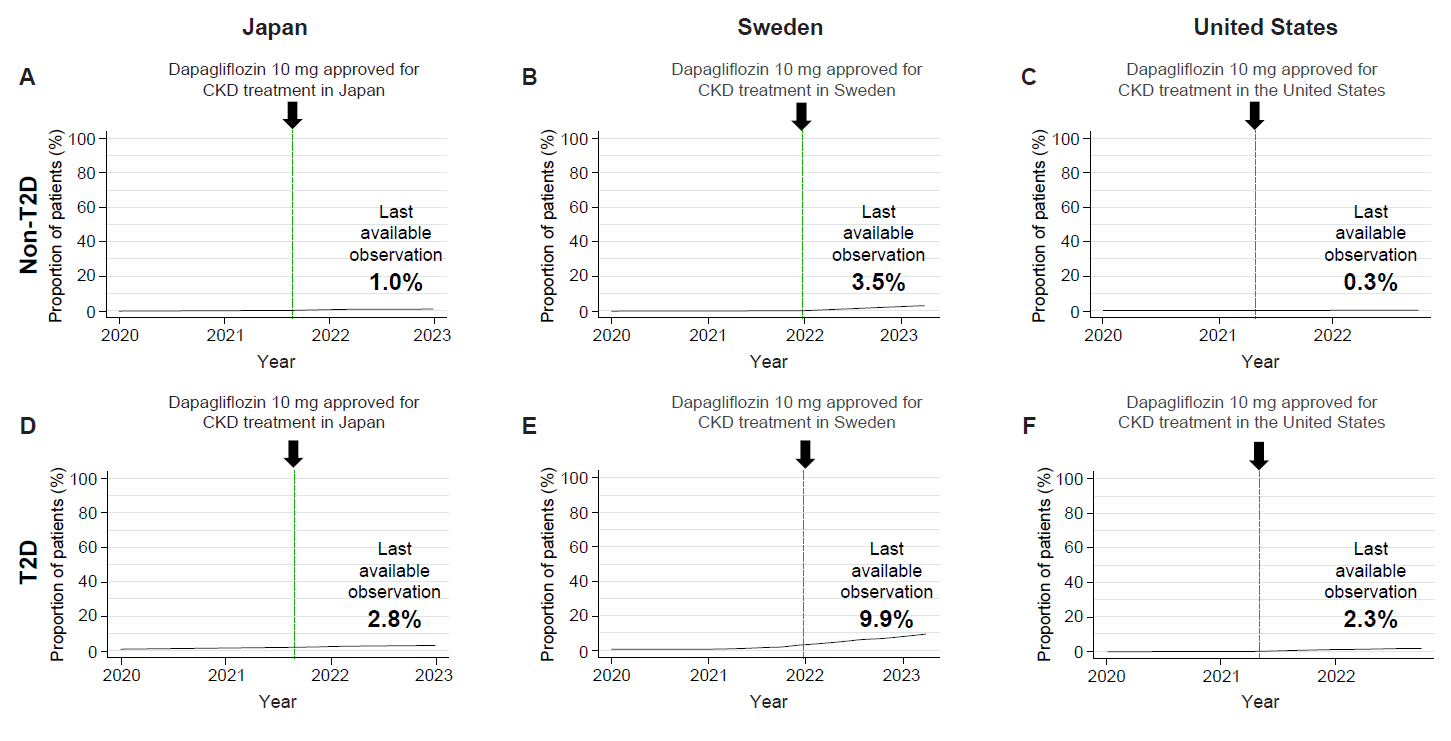


CKD, chronic kidney disease; T2D, type 2 diabetes.

# References

1. Japan Health Policy NOW Health Insurance System, Available at: <https://japanhpn.org/en/hs1/>. Accessed 21 November 2023.

2. Komuro I, Kadowaki T, Bodegard J, Thuresson M, Okami S, Yajima T: Lower heart failure and chronic kidney disease risks associated with sodium-glucose cotransporter-2 inhibitor use in Japanese type 2 diabetes patients without established cardiovascular and renal diseases. *Diabetes Obes Metab,* 23(Suppl 2)**:** 19-27, 2021 10.1111/dom.14119

3. Birkeland KI, Bodegard J, Banerjee A, Kim DJ, Norhammar A, Eriksson JW, et al.: Lower cardiorenal risk with sodium-glucose cotransporter-2 inhibitors versus dipeptidyl peptidase-4 inhibitors in patients with type 2 diabetes without cardiovascular and renal diseases: A large multinational observational study. *Diabetes Obes Metab,* 23**:** 75-85, 2021 10.1111/dom.14189

4. Medical Vision Database, Available at: <https://en.mdv.co.jp/about-mdv-database/>. Accessed 8 July 2022.

5. Kosiborod M, Lam CSP, Kohsaka S, Kim DJ, Karasik A, Shaw J, et al.: Cardiovascular events associated with SGLT-2 inhibitors versus other glucose-lowering drugs: the CVD-REAL 2 study. *J Am Coll Cardiol,* 71**:** 2628-2639, 2018 10.1016/j.jacc.2018.03.009

6. Tanabe M, Motonaga R, Terawaki Y, Nomiyama T, Yanase T: Prescription of oral hypoglycemic agents for patients with type 2 diabetes mellitus: a retrospective cohort study using a Japanese hospital database. *J Diabetes Investig,* 8**:** 227-234, 2017 10.1111/jdi.12567

7. Tanabe M, Nomiyama T, Motonaga R, Murase K, Yanase T: Reduced vascular events in type 2 diabetes by biguanide relative to sulfonylurea: study in a Japanese Hospital Database. *BMC Endocr Disord,* 15**:** 49, 2015 10.1186/s12902-015-0045-y

8. Kohsaka S, Morita N, Okami S, Kidani Y, Yajima T: Current trends in diabetes mellitus database research in Japan. *Diabetes Obes Metab,* 23 Suppl 2**:** 3-18, 2021 10.1111/dom.14325

9. Nishimura R, Kato H, Kisanuki K, Oh A, Onishi Y, Guelfucci F, et al.: Comparison of persistence and adherence between fixed-dose combinations and two-pill combinations in Japanese patients with type 2 diabetes. *Curr Med Res Opin,* 35**:** 869-878, 2019 10.1080/03007995.2018.1551192

10. Norhammar A, Bodegard J, Nystrom T, Thuresson M, Eriksson JW, Nathanson D: Incidence, prevalence and mortality of type 2 diabetes requiring glucose-lowering treatment, and associated risks of cardiovascular complications: a nationwide study in Sweden, 2006-2013. *Diabetologia,* 59**:** 1692-1701, 2016 10.1007/s00125-016-3971-y

11. Ludvigsson JF, Andersson E, Ekbom A, Feychting M, Kim JL, Reuterwall C, et al.: External review and validation of the Swedish national inpatient register. *BMC Public Health,* 11**:** 450, 2011 10.1186/1471-2458-11-450

12. Ludvigsson JF, Otterblad-Olausson P, Pettersson BU, Ekbom A: The Swedish personal identity number: possibilities and pitfalls in healthcare and medical research. *Eur J Epidemiol,* 24**:** 659-667, 2009 10.1007/s10654-009-9350-y

13. Sundstrom J, Bodegard J, Bollmann A, Vervloet MG, Mark PB, Karasik A, et al.: Prevalence, outcomes, and cost of chronic kidney disease in a contemporary population of 2.4 million patients from 11 countries: The CaReMe CKD study. *Lancet Reg Health Eur,* 20**:** 100438, 2022 10.1016/j.lanepe.2022.100438

14. US Department of Health and Human Services Guidance regarding methods for de-identification of protected health information in accordance with the health insurance portability and accountability act (HIPAA) privacy rule, Available at: <https://www.hhs.gov/hipaa/for-professionals/privacy/special-topics/de-identification/index.html>. Accessed 16 August 2023.

15. US Department of Health and Human Services 45 CFR 46, Available at: [www.hhs.gov/ohrp/humansubjects/guidance/45cfr46.html#46.101](file:///C:\Users\Megan.Hein\Downloads\www.hhs.gov\ohrp\humansubjects\guidance\45cfr46.html#46.101). Accessed 16 August 2023.
